# Benznidazole therapy improves pressure overload and cardiac electrical profile in an experimental model of Angiotensin II infusion-induced hypertension: Mechanistic insights

**DOI:** 10.1371/journal.pone.0340280

**Published:** 2026-01-27

**Authors:** Ana Paula da Silva Pinheiro, Glaucia Vilar-Pereira, Leda Castaño-Barrios, Isalira Peroba Rezende Ramos, Yasmin Pedra-Rezende, Luiza Dantas-Pereira, Rubem Figueiredo Sadok Menna-Barreto, Daniel Gibaldi, Hilton Antônio Mata-Santos, Joseli Lannes-Vieira

**Affiliations:** 1 Laboratório de Biologia das Interações, Instituto Oswaldo Cruz/Fiocruz, Rio de Janeiro, Brazil; 2 Departamento de Microbiologia e Parasitologia, Instituto de Biologia, Universidade Federal de Pelotas, Pelotas, Rio Grande do Sul, Brazil; 3 Centro Nacional de Biologia Estrutural e Bioimagem, CENABIO, Universidade Federal do Rio de Janeiro, UFRJ, Rio de Janeiro, Brazil; 4 Laboratório de Biologia Celular, Instituto Oswaldo Cruz/Fiocruz, Rio de Janeiro, Brazil; 5 Faculdade de Farmácia, Universidade Federal do Rio de Janeiro, UFRJ, Rio de Janeiro, Brazil; University of Nebraska Medical Center College of Medicine, UNITED STATES OF AMERICA

## Abstract

High blood pressure is one of the leading global causes of cardiovascular diseases. The chronic action of high concentrations of angiotensin II (Ang II) promotes arterial hypertension. Ang II acts via AT_1_ and AT_2_ receptors. Acting via AT_1_R, Ang II can induce the production of inflammatory cytokines and reactive oxygen species, promoting oxidative stress, which may influence cardiac electrical traits. In hypertensive patients, a dispersed QTc interval may predict cardiovascular events and mortality. Benznidazole (Bz), an antiprotozoal prodrug, also has immunomodulatory properties. Here, we tested the idea that in a model of Ang II-induced BP overload, cardiomyopathy will be associated with a prolonged QTc interval. Then, we investigated the effects of Bz therapy on BP overload, electrical changes, and oxidant/antioxidant imbalance. C57BL/6 mice were implanted with an osmotic minipump containing Ang II or saline as a control. At 7 days post-surgery (dps), Ang II infusion increased mean BP, which was sustained until 28 dps. Further, the Ang II-infused group had prolonged QTc interval and QRS complex. Bz or the AT_1_R antagonist losartan (Los) were administered from 7 to 28 dps. Compared with the vehicle-treated group, Los therapy restored mean BP to normal but did not affect long-QTc. At 14 and 28 dps, Bz therapy improved BP, and restored QTc dispersion to normal, while improving RR interval and QRS complex changes. Ang II infusion increased IL-6 concentrations and oxidant/antioxidant imbalance in cardiac tissue. Bz therapy showed a beneficial effect, tending to restore the IL-6 concentrations and oxidant/antioxidant balance to physiological levels, which was correlated with reversal of the dispersed QTc interval. Altogether, our data support that Bz therapy deserves further evaluation as an anti-inflammatory and antioxidant adjuvant tool to improve BP overload and long-QTc syndrome underlying cardiovascular diseases.

## Introduction

High blood pressure (BP) is a risk factor for the onset and progression of cardiovascular diseases. These are the leading causes of death worldwide, 1.4 billion adults aged 30–79 worldwide had hypertension, in 2024 [1]. BP represents one of the greatest challenges to global public health due to its high prevalence and the cardiovascular complications it causes. Pressure overload triggers adaptive remodeling, which may or may not lead to heart failure [2]. Furthermore, this hemodynamic overload can interfere with the function of ion channels and repolarization processes, compromising the stability of cardiac electrical activity [3]. Moreover, cardiovascular diseases are expected to cause 12% to 22% of deaths worldwide by 2050 [1].

The renin-angiotensin-aldosterone system (RAAS) is critical for cardiovascular physiology, regulating vascular tone and BP homeostasis [4,5]. Angiotensin II (Ang II), a key effector peptide of the RAAS, controls BP through mechanisms involving vasoconstriction and aldosterone release. However, the continuous release of elevated levels of Ang II promotes chronic arterial hypertension, cardiac inflammation, and electrical alterations such as atrial fibrillation [6,7]. Hemodynamic and non-hemodynamic effects of Ang II result from the binding of Ang II to its two receptors, type 1 (AT_1_R) and type 2 (AT_2_R). Ang II binding to AT_1_R promotes activation of signaling pathways involving NF-kB and MAPK, leading to biological consequences such as inflammation and increased release of reactive oxygen species (ROS) [8,9]. The mechanistic influence of Ang II on cardiac electrophysiological properties remains to be elucidated and may involve ROS release and chronic inflammation [10]. Furthermore, cardiovascular diseases may promote cardiac electrical changes, including the prolonged heart rate-corrected QT (QTc) interval syndrome, a predictor of adverse cardiac events and mortality in hypertensive patients [11]. The long-QTc syndrome may be triggered by inflammation involving increased expression of cytokines such as interleukin (IL) −6 and tumor necrosis factor (TNF), and oxidative stress [12]. Indeed, in a preclinical model of Ang II-induced cardiac inflammation in C57BL/6 mice, increased BP is associated with elevated production of TNF in the cardiac tissue [13,14].

The onset and progression of cardiovascular diseases associated with arterial hypertension are triggered by a cascade of events, such as the chronic high levels of Ang II, which promote BP overload. Numerous classes of medications are available aimed at reducing these effects, such as angiotensin receptor blockers (ARB) [15]. Losartan (Los) is a selective competitive AT_1_R antagonist prescribed to patients with hypertension associated or not with cardiac hypertrophy and heart failure [16]. Los promotes a decrease in BP overload, although part of the hypertensive patients are non-responsive to this therapy [17]. Further, new therapies aiming at reducing the side effects (hyperkalemia, nausea, headaches) of the existing therapies, allowing greater adherence to treatment, and focusing on hypertensive patients with refractory hypertension are a current demand [18]. Considering that chronic BP overload can contribute to inflammation and oxidative stress [19], processes mechanistically underlying cardiovascular diseases [12] became the focus of our study, exploring the repositioning of a therapeutic strategy.

Benznidazole (Bz) is a prodrug used for etiological treatment of the protozoan parasite *Trypanosoma cruzi*, the causative agent of Chagas disease [20]. Bz therapy has beneficial effects reducing cardiovascular events and mortality when used in chronic patients without severe cardiopathy [21]. In preclinical Chagas disease models, Bz therapy shows beneficial effects on electrical changes restoring prolonged QTc interval, and immunomodulatory properties controlling levels of inflammatory cytokines and oxidative stress in tissues and systemically [22–25]. These beneficial effects are commonly considered secondary to the reduction in stimulation following parasite control. Nonetheless, the immunomodulatory effects of Bz therapy are also observed in a model of LPS-induced sepsis, associated with downregulation of TNF production, while favoring antioxidant pathways [26,27].

Together, these findings led us to use an early-phase Ang II infusion-induced hypertension model with preserved cardiac structure and function [13,28] to investigate the effects of Bz therapy on BP and cardiac electrical changes, particularly on dispersed QTc intervals. Further, trying to bring mechanistic insights, we analyzed the serum cytokine levels, as a readout of systemic inflammatory profile, and cytokine and oxidative stress in cardiac tissue, and their association with clinical changes.

## Methods

### Ethics statement

The experimental procedures were performed in accordance with the Fiocruz Animal Use Ethics Committee (CEUA/Fiocruz), through protocols approved (CEUA–IOC L006-2018-A3 and L002-2023-A2 Licenses), and conducted according to the Brazilian Federal Law 11.794 (October 8th, 2008). Female mice of the C57BL/6 lineage aged 8–10 weeks were provided by the Institute of Science and Technology in Biomodels (ICTB-Fiocruz) and maintained in the Animal Facility of IOC/Fiocruz. All data were obtained from three independent experiments (Register Books #75 and #79, LBI/IOC-Fiocruz).

### Experimental design

35 animals were randomly distributed in experimental groups as shown in the workflow (S1A Fig) and subjected to the proposed therapeutic strategies (S1B Fig). Mice were lodged in numbered cages, marked on the ears with an ear punch, according to a pre-established code, receiving water and grain-based food *ad libitum*. To minimize the effects of stress and allow the adaptation process to the new environment, mice were kept without manipulation for 14 days in cages, provided with environmental enrichment (igloo). After the adaptation period, the experiments were carried out (S1A Fig), according to the described therapeutic schemes (S1B Fig). In Experiment 1 (Exp. 1), four animals were infused with saline and six with Ang II and were analyzed at 28- and 58-day post-surgery (dps). In Exp. 2, three mice were part of the control group (saline), and animals infused with Ang II were divided into three experimental groups: mice treated with vehicle (non-pyrogenic water; Veh; n = 3), or with the AT_1_R blocker losartan (Los; n = 3) or with benznidazole (Bz; n = 3). In Exp. 3, four mice were infused with saline, and nine mice were infused with Ang II and divided into two experimental groups: treated with Veh (n = 5), or with Bz (n = 4). At 28 and 58 dps, after topical eye drops anesthesia (tetracaine hydrochloride 1%, phenylephrine hydrochloride 0.1%), mice were bled by the orbital plexus to obtain serum. Mice were euthanized at the endpoints (28 and 58 dps) using CO_2_ inhalation in an appropriate chamber, allowing 70% CO_2_ saturation for 2–3 minutes.

### Osmotic minipump implant

All procedures were carried out as previously described [13]. Osmotic minipumps (Pump model 1004, Alzet, Lab Research, USA) containing 100 µL of saline (Sal) or 100 µL of saline containing 2 mg/Kg/day of Ang II (Sigma, A6402-5MG, USA), with a pumping rate of 0.11 µL/hour and lasting capacity of 28 days, were prepared according to manufacturer’s recommendations. On the day of surgery for the minipumps implantation, mice were sedated and anesthetized with a combination of Xylazine (100 mg/Kg) and Ketamine (8 mg/Kg). The minipumps were implanted subcutaneously in the dorsal region between the scapulae, according to the manufacturer’s procedures (https://www.jove.com/v/53191/subcutaneous-angiotensin-ii-infusion-using-osmotic-pumps-induces). After surgery, the mice were monitored at the same time every day, applying a topical analgesic, such as lidocaine, and a topical antibiotic (5 mg neomycin sulfate and 250 IU bacitracin zinc/g) to the surgical area for 6 days. Weekly, clinical aspects, such as body weight, were evaluated.

### Treatments

Seven days after the beginning of the infusion, the mice were subjected to a daily therapeutic regimen via oral gavage with 100 μL of pyrogen-free water (Veh; BioManguinhos/Fiocruz), which continued until the 28^th^ dps, totaling 21 days of therapy. The mice infused with saline received Veh. According to the experimental designs, the Ang II-infused mice were divided into three groups: Veh group, Bz group (25 mg/Kg/day; LAFEPE, Brazil), and Los group (10 mg/Kg/day; Eurofarma, Brazil).

### Blood pressure analysis

A two-channel tail-cuff plethysmography system (Bonther, Brazil) was used to assess blood pressure. Prior to blood pressure measurement, the mice were acclimated to the experimental environment for three consecutive days. This acclimation process involved placing the mice daily at the same time in a restrainer for approximately two minutes to reduce stress and heart rate variability. On the day of measurement, the mice were placed in a bio-heater (EFF 307, Insight, Brazil) at 37°C for 5 minutes to promote vasodilation of the caudal artery. Immediately afterward, the mice were placed in the restrainer with their tails positioned on the heated base, and the cuffs were placed on the mice’s tails and aligned with the optical sensor, which detects changes in the tail blood vessels. In our study, we standardized two acclimation cycles for mice to adapt to the new stressor environment, followed by three valid measurement cycles, detecting systolic, diastolic, and mean arterial pressure. The data were exported to an Excel spreadsheet and subsequently analyzed.

### Electrocardiographic evaluation

To evaluate electrical conduction, electrocardiogram (ECG) recorders were registered on day 0 (pre-surgery), 14 and 28 dps. All mice were previously tranquilized with intraperitoneal Diazepam (10 mg/Kg), and the electrons were placed subcutaneously, according to the DII derivation. Data were recorded for 2 minutes by detecting the traces in the digital system (Power Lab 20/02) connected to a 2mV amplifier for 1 s (PanLab Instruments, Spain), filters were stabilized between 0.1 and 100 Hz, and the tracings were acquired. The RR, PR, QRS and QT intervals, and the QRS complex were manually measured from ECG traces and expressed as milliseconds (ms). The QT interval was measured from the onset of the QRS complex to the end of the T wave (return to the isoelectric baseline) and corrected for heart rate using Bazett’s formula (QTc). Data analysis was performed using PowerLab equipment, LabChart version 7 PRO software, as previously described [22].

### Echocardiographic study

To analyze cardiac function, an echocardiogram (ECHO) was performed on the 28^th^ dps. The animals were anesthetized using 1.5% isoflurane gas in oxygen at a flow rate of 1 liter/minute and trichotomized in the precordial region to evaluate the parameters on the Vevo 770 device (Visual Sonics, Canada) connected to a 30 MHz transducer. Images in two-dimensional mode (Mode B) were used to analyze the right and left ventricular areas. M-Mode imaging was used to assess ventricular thickness. Left ventricular ejection fraction (LVEF%) and fractional area change (FAC%) were determined using the Simpson’s method [29].

### Heart extracts preparation

The collected and frozen cardiac tissues were weighed and cut. Tissues of 10–15 mg were obtained for extraction. The tissues were slowly washed 2–3 times with 1 mL of PBS. For each 5 mg of tissue, 300 µL of Ripa Buffer (Sigma, USA) containing the protease inhibitor cocktail at a dilution of 1/10 was added, and the mixture was stored at 4 °C. The tissues contained in the mixture of RIPA buffer and protease inhibitor were safely placed on ice and homogenized in a tissue grinder (Turraz T10 BS1-5G, IKA, Germany) in a cryotube at 3000 rpm (Position 6) for 10 seconds, with 10-second rest intervals, repeating the entire cycle 6 times. After the homogenization cycles, the contents were shaken for 2 hours at 4 °C. Finally, the samples were transferred to a 1.5 mL Eppendorf tube and centrifuged at 13600 G (200R Hettich Mikro Centrifuge, Germany), for 20 minutes at 4 °C, and the supernatants were collected, aliquoted and co-frozen at −80 °C for later studies, a previously described protocol [26].

### Determination of cytokines in sera and heart extracts

The levels of cytokines in serum and heart extracts were measured using the BD Cytometric Bead Array (CBA) Mouse Th1/Th2/Th17 CBA Kit (catalog 560485, BD Bioscience, USA). The kit was used for the simultaneous detection of IL-2, IL-4, IL-6, interferon-γ (IFN-γ), TNF, IL-17A, and IL-10 protein levels in a single sample. The protocol was carried out according to the manufacturer’s recommendations. Cytokine standards were diluted serially to construct the calibration curves and used to determine the cytokine concentrations. The samples were analyzed using the 13-Color CytoFLEX-S flow cytometer (Beckman-Coulter, USA). Individual cytokine concentrations were indicated by their fluorescent intensities and expressed in pg/mL, using the FCAP Array Software. The theoretical limits of detection were: 0.1 pg/mL for IL-2, 0.03 pg/mL for IL-4, 1.4 for IL-6, 0.5 pg/mL for IFN-γ, 0.9 pg/mL for TNF, 0.8 pg/mL for IL-17A, and 16.8 pg/mL for IL-10.

### NADP/NADPH evaluation

For the analysis of NADP/NADPH in cardiac extracts, the assay was performed according to the manufacturer’s instructions. Briefly, a standard curve was obtained by serial dilutions of a NADPH solution. In a microplate, 50 μL of the NADP/NADPH reaction mixture was added to each well of the NADPH standard, control, blank (PBS), and test samples to make the total volume of the NADP/NADPH assay 100 μL/well. The reaction was incubated at room temperature for 15 minutes to 2 hours, protected from light, and the absorbance at a wavelength of 460nm was measured in the spectrophotometer (SpectraMax M3 Plus, USA).

### Detection of reactive oxygen species

The hearts were collected after 28 dps, sectioned, embedded in tissue-freezing medium (Tissue-Tek, Miles Laboratories, USA), and stored in liquid nitrogen. For analysis of oxidative stress, serial 3-μm-thick cryostat sections of the ventricles were obtained and used to evaluate the production of ROS in heart tissue by staining unfixed tissue with 50 mM dihydroethidium (DHE, D11347, Thermo Fisher, USA) for 30 minutes. After incubation, the sections were fixed in a 4% paraformaldehyde solution. Images were obtained using a Zeiss Axio Observer M1 (Zeiss, Oberkochen, Germany) microscope. The analysis of acquired images was performed using NIS Elements software (Nikon Co., Japan) with ten fields per mouse heart sample [25]. DHE analysis was performed on ten microscopic fields per slice (2 slices per mouse), ensuring representativeness and consistency in the quantification of fluorescence and are associated with the production of reactive oxygen species.

### Assessment of lipid peroxidation

To assess lipid peroxidation, the presence of thiobarbituric acid-reactive substances (TBARS), products of degradation of hydroperoxides and lipid peroxides formed during the oxidation of fatty acids, was determined in heart extracts, as previously described [30]. Aliquots of 50 μL of the tissue extracts were incubated in a dry bath with 12 μL of a medium containing 8.1% sodium dodecyl sulfate (SDS), 94 μL glacial acetic acid (99.8%), and 94 μL of 0.6% thiobarbituric acid (TBA) at a temperature of 100 °C for 60 minutes. After heating the mixture, the samples are centrifuged at 3000 rpm for 10 minutes at 4 ºC to avoid thermal degradation and improve separation. The supernatant was collected with the TBA complex. The reaction product was read in a spectrophotometer (SpectraMax I3, Molecular Devices, Austria) at a wavelength of 532 nm [24].

### Measurement of SOD activity

To evaluate the enzymatic activity of superoxide dismutase (SOD), 50 μL of the tissue extracts were added to 50 μL of the reaction mixture containing 50 mM potassium phosphate buffer, 0.13 mg/mL BSA (A3803; Sigma, USA), 1 U/mL catalase (C9322; Sigma, USA), 100 μM xanthine (1196-43-6; Sigma, USA), 55 μM nitrotetrazolium blue (NBT) (298-83-9; Sigma, USA) and 0.1 U/mL xanthine oxidase (9002-17-9; Sigma, USA). The NBT reduction was read on a spectrophotometer (SpectraMax M3 Plus, Molecular Devices, USA) at a wavelength of 560 nm [31].

### Statistical analysis

All data are shown as mean ± standard deviation (SD). To assess the normality of the data, the Shapiro-Wilks tests were used. For two-group analysis, the Student’s T test with a 95% confidence level was used for data with normal distribution, and the Mann-Whitney test for data without normal distribution. The One-Way test (ANOVA) associated with the Tukey post-test was used to compare more than two groups. For correlation analysis we used Pearson correlation coefficient and two-tailed test. Analyses were performed using GraphPad Prism 8.0.1 (La Jolla, CA, USA). Differences were considered statistically significant when *p* < 0.05.

## Results

### Electrical but not functional alterations are detected in a model of Ang II-promoted blood pressure overload in C57BL/6 mice

**Fig 1:**
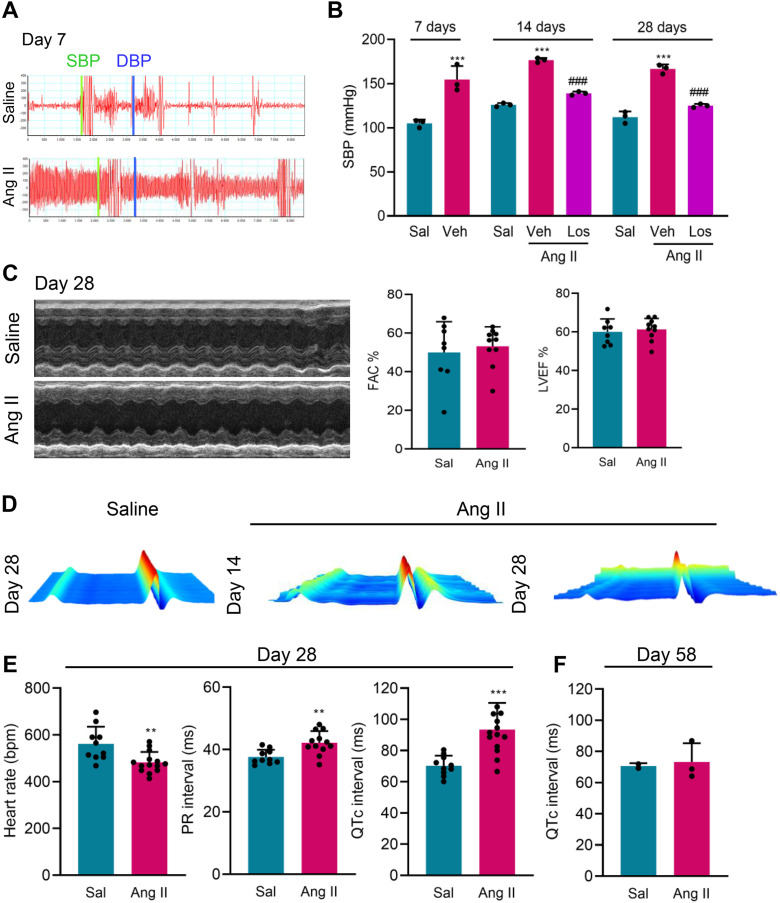
Infusion with Ang II (2 mg/Kg/day) promotes blood pressure overload in parallel with prolonged QTc. (A) Representative images of blood flow, SBP (green line) and DBP (blue line) in Sal-infused and Ang II-infused C57BL/6 mice, at 7 dps. (B) The graph shows systolic blood pressure (SBP) in Sal-infused and vehicle (Veh) and losartan (Los) treated Ang II-infused C57BL/6 mice. Mice were analyzed at 7 (pre-therapy), 14 and 28 dps. (C) Representative images of the ventricular chamber of Sal- and Ang II-infused mice, at 28 dps. Graphs show FAC% and LVEF%, evaluated according to Simpson’s method. (D) Representative images of the 3D ECG tracing recorded at 14 and 28 dps. (E) Graphs show heart rate (beats per minute, bpm), PR interval duration (ms), and QTc interval (ms), at 28 dps. (F) The graph shows QTc interval (ms) evaluated 30 days after the end of the Ang II infusion (58 dps). Data are shown as means ± SD. ^**^, p < 0.01, ^***^, p < 0.001, Sal *vs* Ang II + Veh; ^###^, p < 0.001, Ang II + Veh *vs* AngII + Los (test: ANOVA, post-test: Tukey’s multiple comparisons).

S1 Table shows data used to build graphs and figures. In experiment 1, we established a BP overload model using female C57BL/6 mice implanted with an osmotic minipump containing Ang II, compared with Sal infusion as a control. All mice subjected to pump implantation survived (S2A Fig). At 28 and 58 dps, Ang II infusion led to a 2–3% loss of initial body weight, while Sal-infused mice gained weight (S2B Fig). During the experimental time course, the body weights of Veh- and Bz-treated Ang II-infused groups had compromised weight gain (p < 0.01) compared with Sal group (S2C Fig). At 7 dps, compared with Sal-infused mice, Ang II-infused mice showed an increase in systolic BP (SBP; p < 0.001; Fig 1A, 1B), diastolic BP (DBP; Sal, 128 ± 20 mmHg *vs* Ang II, 175 ± 13 mmHg; p < 0.01), and mean BP (MBP; Sal, 114 ± 12 mmHg *vs* Ang II, 156 ± 13 mmHg; p < 0.001), as shown in Table 1. Consistent with the pumping capacity of Ang II release, these changes persisted after 14 dps (p < 0.001) and 28 dps (p < 0.001) (Fig 1B). Compared with Veh treatment, Ang II-infused mice treatment with the AT_1_R antagonist Los reduced the elevated SBP (p < 0.001), as shown in Fig 1B. These results are seen at 28 dps, evaluating SBP (Sal, 132 ± 10 mmHg *vs* Ang II + Veh, 167 ± 12 mmHg *vs* AngII + Los, 125 ± 3 mmHg; p < 0.001), DBP (Sal, 112 ± 8 mmHg *vs* Ang II + Veh, 148.6 ± 14 mmHg *vs* AngII + Los, 117 ± 3 mmHg; p < 0.001), and MBP (Sal, 117 ± 10 mmHg *vs* Ang II + Veh, 156.8 ± 10 mmHg *vs* AngII + Los, 124 ± 4 mmHg; p < 0.001), as shown in Table 1.

**Table 1:** Bz therapy ameliorates clinical parameters.

		Groups (Mean ± SD)		
Parameters	Days post-surgery	Saline	Ang II + Veh	Ang II + Bz
Diastolic blood pressure (mmHg)	7	106.8 ± 8	153.5 ± 14^***^	
	14	109.3 ± 13	146.6 ± 15^***^	134.4 ± 9^*^
	21	104.3 ± 6	137.6 ± 4^***^	112.7 ± 6^##^
	28	110.1 ± 6	153.1 ± 13^***^	134.8 ± 10^*,#^
Systolic blood pressure (mmHg)	7	128 ± 20	175 ± 13^**^	
	14	128.6 ± 14	168 ± 18^***^	153 ± 12^*^
	21	123.6 ± 1	155.9 ± 6^***^	130.8 ± 5^##^
	28	132.2 ± 10	167 ± 12^***^	146.1 ± 23^*,#^
Mean blood pressure (mmHg)	7	114.2 ± 12	155.9 ± 10^***^	
	14	115.6 ± 13	156.3 ± 13^***^	136.2 ± 10^*,#^
	21	112.9 ± 7	143.7 ± 4^***^	118.7 ± 6^##^
	28	114.4 ± 7	157.8 ± 7^***^	138.7 ± 20^*,#^
Heart rate (bpm)	0	556 ± 93		
	14	545 ± 100	492.1 ± 49.7^&&&, *^	482.1 ± 65.7
	28	561 ± 73.1	482.2 ± 44.6^&&&, *^	580.3 ± 32
PR interval (ms)	0	37.8 ± 2.95		
	14	39.1 ± 2.8	42.2 ± 2.6^&&^	41.7 ± 3.6^&^
	28	38.7 ± 2.34	42.3 ± 2.7^&&^	39.8 ± 2.3
RR interval (ms)	0	112.1 ± 24		
	14	105 ± 12	123.5 ± 13	126.2 ± 18
	28	108. 3 ± 13	124.8 ± 9.8	103.6 ± 5.7
QRS complex (ms)	0	10.91 ± 1		
	14	11.23 ± 1	13.32 ± 1^&^	12 ± 1
	28	10.47 ± 0.8	13.28 ± 1^&, *^	12 ± 0.01
QTc interval (ms)	0	67.30 ± 4.3		
	14	75.24 ± 2.6	106.4 ± 3.6^&&&, ***^	79.57 ± 17^#^
	28	70.71 ± 6.4	114.8 ± 19^&&&, ***^	83.67 ± 10^#^

* , p < 0.05; ** , p < 0.01;*** , p < 0.001, Sal *vs* Ang II infusion;

# , p < 0.05; ^##^, p < 0.01; Ang II + Veh *vs* Ang II + Bz;

& p < 0.05; ^&&^, p < 0.01; ^&&&^, p < 0.001, day 0 (pre-surgery) *vs* Ang II + Veh or Ang II + Bz;

Test: ANOVA, post-test: Tukey’s multiple comparisons.

At 28 dps, ECHO evaluation showed that cardiac functionality was preserved in Ang II-infused C57BL/6 mice with FAC% and LVEF% similar to Sal-infused mice (Fig 1C). Together, these data support that the model of BP overload in the absence of structural alterations has been established, reproducing the model previously described [28], therefore allowing the testing of our ideas.

ECG kinetic analysis showed electrical abnormalities in this Ang II-induced BP overload model, at 14 and 28 dps (Fig 1D). At 28 dps, Ang II infusion induced bradycardia (p < 0.01), increased PR interval (p < 0.01) and QTc interval dispersion (p < 0.001), compared with Sal infusion (Fig 1E). As expected, these effects were dependent on the continuous Ang II infusion, as 30 days after the end of infusion (58 dps, S3A Fig), ECG parameters, including bradycardia and prolonged QTc intervals, were restored to normal values (Fig 1F, S3B Fig). Further, in Ang II-infused mice, the relative weight was restored to values found in Sal-infused mice (S3C Fig), and the LVEF% was preserved (S3D Fig) at 58 dps. Therefore, in this BP model of overload, changes in ventricular repolarization with prolonged QTc are dependent on the chronic and continuous Ang II infusion.

### Benznidazole ameliorates blood pressure overload established by Ang II infusion

**Fig 2:**
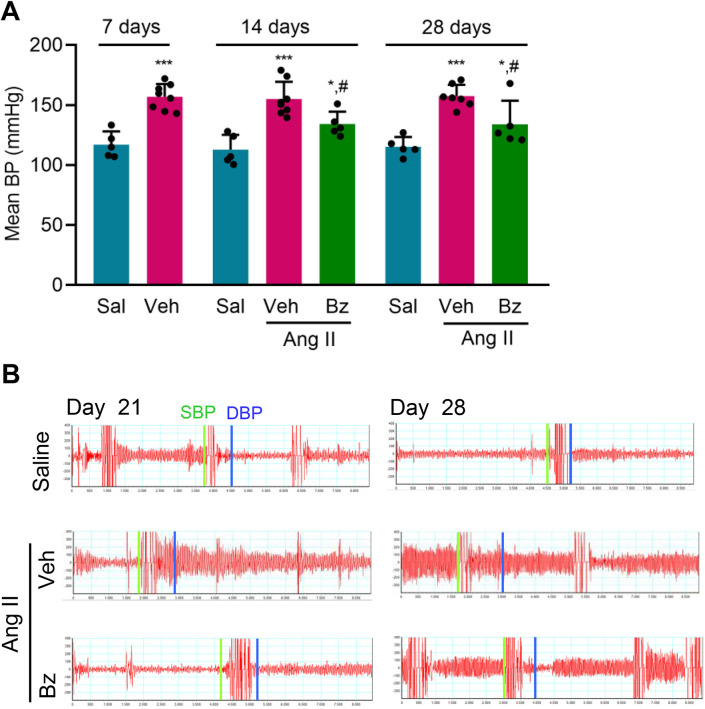
Effect of Bz therapy after Ang II infusion (2 mg/Kg/day) on pressure overload. (A) The graph shows the mean blood pressure (MBP) in Sal-infused, Veh-treated, and Bz-treated Ang II-infused C57BL/6 mice analyzed at 7 (pre-therapy), 14, and 28 dps. (B) Representative images of blood flow and systolic blood pressure (SBP, green line) and diastolic blood pressure (DBP, blue line), at 21 dps and 28 dps. Data are shown as means ± SD. ^*^, p < 0.05, ^***^, p < 0.001, comparison with Sal-infused mice; ^#^, p < 0.05, Ang II + Veh *vs* Ang II + Bz (test: ANOVA, post-test: Tukey’s multiple comparisons).

To test our idea, we evaluated the effect of Bz therapy on the blood pressure overload model induced by infusion of Ang II. In Exp. 2, Bz therapy (25 mg/Kg/day) was initiated at 7 dps, when MBP was elevated in Ang II-infused mice (Fig 2A). The therapeutic scheme was used for 21 consecutive days, until the end of the osmotic minipump operation, at 28 dps (S1B Fig). Beneficial effects of Bz therapy on BP overload were noticed from 14 to 28 dps, when MBP (Fig 2A), DBP, and SBP (S4A-B Fig) were partially decreased in comparison with Veh-treated Ang II-infused mice. Representative figures of blood flow show the beneficial effects of Bz on BP overload of Ang II-infused mice, at 21 and 28 dps (Fig 2B). The reproducibility of the therapeutic effects of Bz on Ang II-induced BP overload is demonstrated in representative figures of blood flow and graphs of DBP and SBP of the complete kinetic evaluation of Exp. 3 (S4A-B Fig, Table 1).

### Bz therapy normalizes heart rate and maintains cardiac electrical tracings within physiological parameters after Ang II infusion

**Fig 3:**
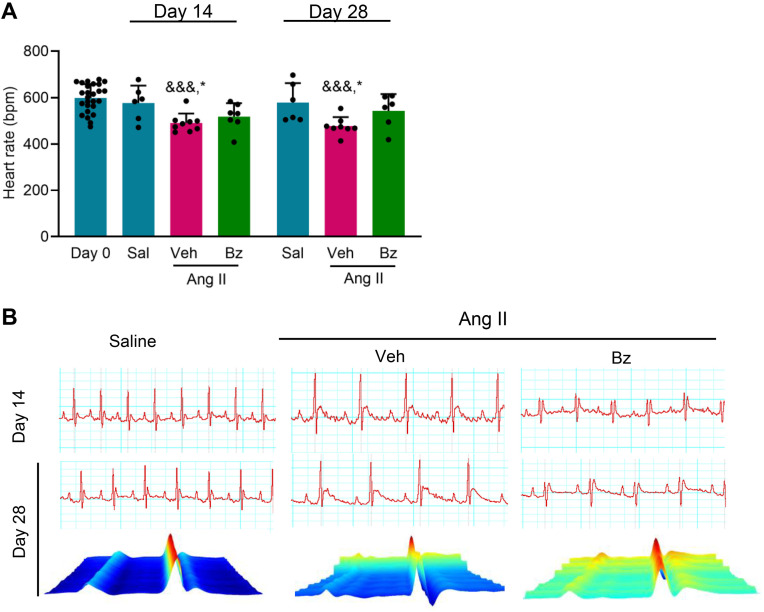
Effects of Bz therapy on heart rate and cardiac electrical activity. (A) Heart rate (bpm) in Ang II-infused mice treated with Veh or Bz compared with day 0 (pre-surgery) and saline-infused group (control), at 14 and 28 days post-osmotic minipump implantation. (B) Representative images of the 2D and 3D tracings of ECG records of saline-infused, Veh-treated, and Bz-treated Ang II-infused C57BL/6 mice analyzed at 14 and 28 dps. Data are shown as means ± SD. ^&&&^ p < 0.001, day 0 *vs* Ang II + Veh; ^*^ p < 0.05, Sal *vs* Ang II + Veh (test: ANOVA, post-test: Tukey’s multiple comparisons).

Next, we challenged the effects of Bz therapy on Ang II-promoted electrical abnormalities, such as the reduced heart rates and increased dispersion of QTc intervals (Fig 1D, 1E). Fig 3A and Table 1 show that Ang II infusion decreased heart rate on days 14 and 28 after mini-osmotic pump implantation, compared with day 0 and the saline-infused group. Bz therapy did not significantly influence heart rate, which was similar to pre-surgery and saline-infused groups (Fig 3A, Table 1). Representative 2D and 3D tracing images of ECG records show that Veh-treated Ang II-infused group exhibits alterations in cardiac electrical conduction, while the group Bz-treated group has patterns similar to the saline-infused group, with a more organized morphology closer to the physiological pattern (Fig 3B). This suggests that Bz therapy may have a beneficial effect on cardiac electrophysiology.

### Benznidazole therapy restores the dispersion of the QTc interval in Ang II-infused mice

**Fig 4:**
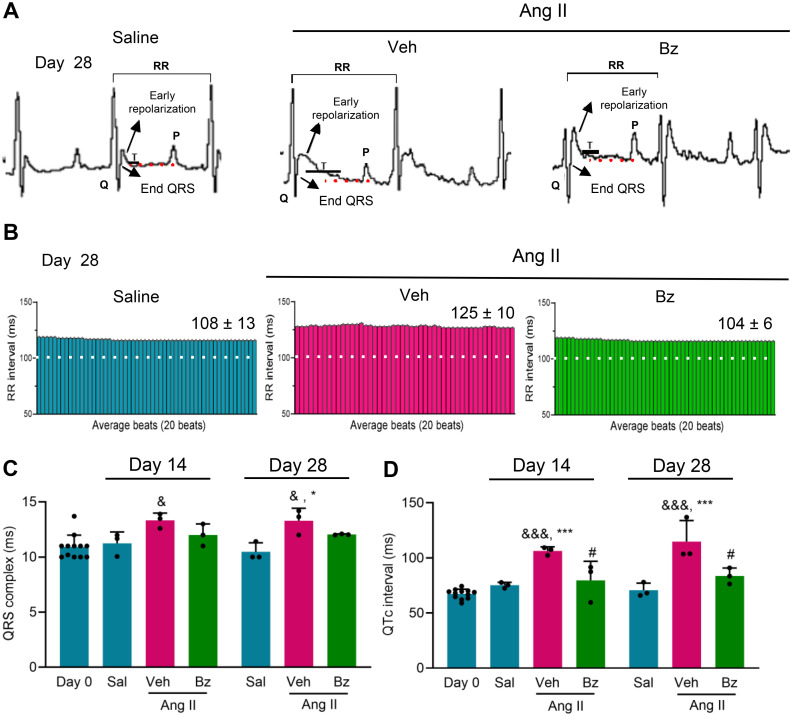
Bz therapy restores the Ang II-induced prolonged QTc interval. (A) Representative images of 2D tracings of ECG records indicating the start and end points (indicated with arrows and dashed red lines) used for QT interval measurement in C57BL/6 mice infused with saline or Ang II, treated with Veh or Bz, analyzed at 28 dps. (B) Representative profiles of RR interval duration (ms) registered in sequential heart beats, at 28 dps. (C) QRS complex duration (ms) in Veh-treated and Bz-treated Ang II-infused mice compared with day 0 (pre-surgery) and the Sal control group, at 14 and 28 dps. (D) QTc interval duration (ms) in Veh-treated and Bz-treated Ang II-infused mice compared with day 0 (pre-surgery) and the Sal control group, at 14 and 28 dps. Data are shown as means ± SD. ^&^ p < 0.05, ^&&&^ p < 0.001, day 0 *vs* Ang II; ^*^ p < 0.05, ^***^ p < 0.001, Sal *vs* Ang II; ^#^ p < 0.05, Veh-treated *vs* Bz-treated Ang II-infused (test: ANOVA, post-test: Tukey’s multiple comparisons).

The analysis of ECG records showed increased PR intervals in Veh-treated Ang II-infused mice, at 14 and 28 dps, compared with the mice pre-surgery (p < 0.01). Beneficial effects of Bz therapy were detected at 28 dps (Table 1). Considering one of our main focuses, we investigated the role of Bz therapy in QT interval dispersion under the influence of pressure overload induced by Ang II infusion. Fig 4A shows representative image of the 2D tracing of the experimental groups indicating the start and end points used for measuring the QT interval, which was measured from the beginning of the QRS complex to the end of the T wave, corresponding to the point where the repolarization phase returns to the isoelectric baseline (Fig 4A). The analysis of the RR intervals per average beat rate revealed a regular pattern, without variations, in all analyzed mice, independently of the infusion (Sal *vs* Ang II) and the therapy (Veh *vs* Bz) used (Fig 4B). However, Ang II-infused mice showed a longer RR interval duration per average beat rate compared with the Sal-infused group, while in Bz-treated Ang II-infused mice, the RR interval duration was preserved (108 ± 13 ms in Sal-infused mice *vs* 125 ± 10 ms in Ang II + Veh *vs* 104 ± 6 ms in Ang II + Bz mice, at 28 dps; Fig 4B, Table 1). Further, Veh-treated Ang II-infused mice showed a prolonged QRS complex compared with the group before surgery (day 0 *vs* Ang II + Veh, at 14 and 28 dps; p < 0.05) or the Sal-infused control group (p < 0.05, at 28 dps). Notably, Bz-treated Ang II-infused mice showed a QRS complex mean duration comparable to the Sal-infused group, at 14 and 28 dps (Fig 4C, Table 1). Moreover, infusion with Ang II promoted an increase in the dispersion of the QTc interval compared with the group pre-surgery (day 0 *vs* Ang II + Veh, at 14 and 28 dps; p < 0.001) and Sal-infused control mice (Sal *vs* AngII + Veh, at 14 and 28 dps; p < 0.001), as shown in Fig 4D. Moreover, Bz-treated Ang II-infused mice showed a decrease in the prolonged QTc interval, compared with the Veh-treated Ang II-infused group (p < 0.05), at 14 and 28 dps (Fig 4D, Table 1).

### Benznidazole therapy does not interfere with cardiac structure and functionality in the model of Ang II-induced pressure overload

**Fig 5:**
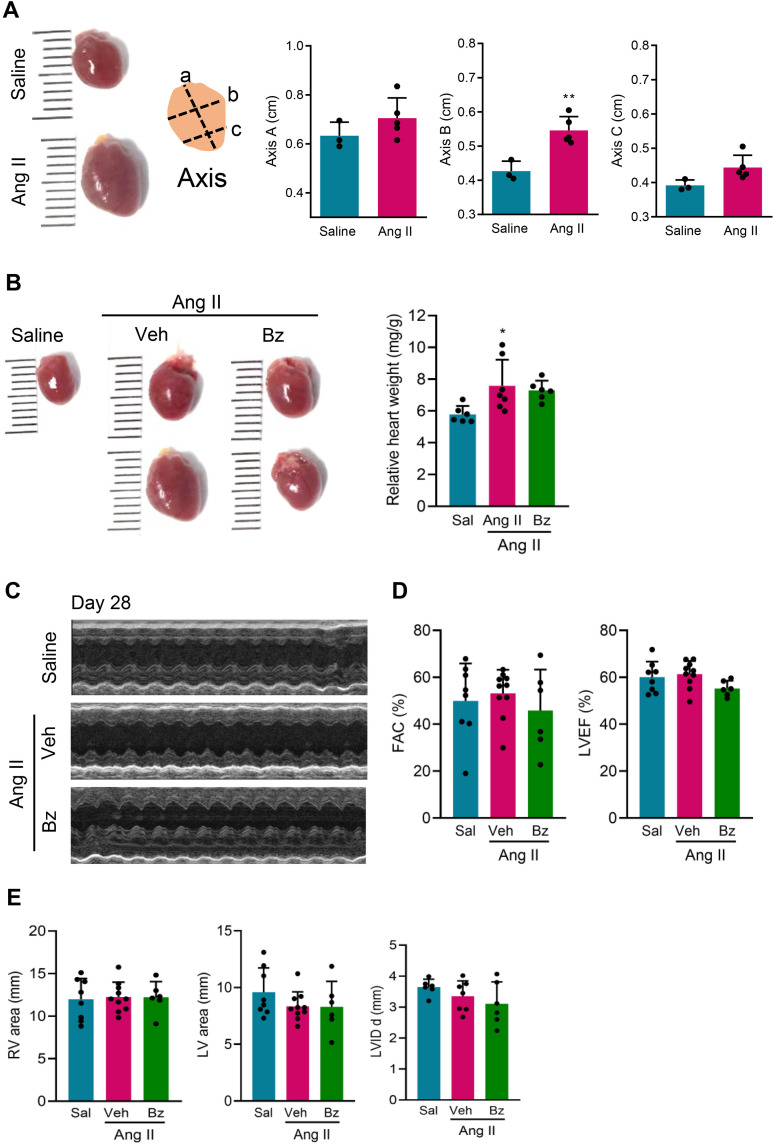
The cardiac function and structure were preserved in benznidazole-treated Ang II-infused mice. (A) Representative images of hearts and graphs showing their dimensions (a, b, and c axis), at 28 dps. (B) Representative images of hearts and relative heart weights, at 28 dps. (C) Representative images of the cardiac ventricular chamber visualized by ECHO analysis, at 28 dps. (D) FAC% and LVEF%, at 28 dps. (E) Right ventricular (RV), left ventricular (LV) areas and internal dimension of the ventricles (LVID), at 28 dps. Data are shown as means ± SD. ^*^, p < 0.05, ^**^, p < 0.01, Sal-infused *vs* Veh-treated Ang II-infused group (test: *t*-Studen*t*; ANOVA, post-test: Tukey’s multiple comparisons).

Ang II infusion promotes hemodynamic imbalance, such as pressure overload, which can lead to increased afterload, resulting in a possible process of adaptive remodeling of the heart, which can be reflected in increased relative weight and changes in functionality [32]. Fig 5A shows that Ang II infusion for 28 days in C57BL/6 mice increased the dimensions of the heart, as evident in axis B (Fig 5A), and augmented the relative heart weight compared with Sal infusion (p < 0.05; Fig 5B). Bz administration to Ang II-infused mice did not change cardiac size and relative heart weight, when compared with the group that received infusion with Sal or with Veh-treated Ang II-infused mice (Fig 5A, 5B). In addition, the hearts of Veh-treated Ang II-infused mice were more dilated and flaccid than the hearts of Sal-infused mice, while the hearts of Bz-treated Ang II-infused mice were more similar to the hearts of Sal-infused control mice (Fig 5A, 5B). At 28 dps, ECHO analysis showed preserved heart structure and function. Representative M-mode images show preserved cardiac chambers in all experimental groups (Fig 5C). The right ventricular functionality revealed by FAC% was preserved in all analyzed groups (Fig 5D, Table 2). The LVEF%, describing the left ventricular systolic function, was preserved in all mice groups (Fig 5D, Table 2). In all studied groups no structural changes were detected, with right and left ventricular areas, and internal dimension of the ventricles (LVID; Fig 5E, Table 2). Further, posterior wall of the LV (LVPW), intraventricular septum (IVS), LV mass corrected, transmitral flow velocity during ventricular filling (MV E), transmitral flow velocity during atrial contraction (MV A), time integral and pulmonary flow velocity (PV VTI), systolic volume, diastolic volume and stroke volume (SV) were similar in all studied groups (Table 2). Thus, Bz therapy does not interfere with cardiac function and structure providing confidence in its safety and lack of promotion of ventricular dysfunction in Ang II-infused in C57BL/6 mice, at 28 dps (Table 2).

**Table 2:** ECHO parameters analyzed at 28-days post-surgery.

	Groups (Mean ± SD)		
Parameters	Saline	Ang II + Veh	Ang II + Bz
FAC (%)	49.9 ± 15.9	53.14 ± 10.1	45.81 ± 17.5
LVEF (%)	60 ± 6.66	61.3 9 ± 5.69	55.25 ± 3.29
RV area (mm)	11.99 ± 2.43	12.25 ± 1.75	12.21 ± 1.85
LV area (mm)	9.59 ± 2.14	8.35 ± 1.27	8.29 ± 2.25
LVID d (mm)	3.64 ± 0.26	3.35 ± 0.50	3.11 ± 0.71
LVPW, d (mm)	0.83 ± 0.07	1.02 ± 0.18	1.03 ± 0.30
IVS,d (mm)	0.84 ± 0.12	0.96 ± 0.15	1.04 ± 0.25
LV Mass corrected (mg)	86.38 ± 16.95	94.75 ± 11.72	89.61 ± 16.05
MV E (mm/s)	514.76 ± 172.17	419.20 ± 150.75	469.76 ± 117.04
MV A (mm/s)	274.33 ± 93.33	255.95 ± 98.52	236.73 ± 36.17
MV E/A (mm/s)	1.96 ± 0.68	1.66 ± 0.31	2.01 ± 0.53
PV VTI (cm)	3.46 ± 0.22	2.97 ± 0.56	2.74 ± 0.68
Systolic volume (µL)	21.34 ± 4.74	19.06 ± 4.58	24.30 ± 6.75
Diastolic volume (µL)	56.55 ± 8.16	52.65 ± 11.06	54.03 ± 12.68
SV (%)	35.20 ± 7.01	33.60 ± 7.16	29.73 ± 6.38

Test: ANOVA, post-test: Tukey’s multiple comparisons.

### Beneficial effects of Bz therapy on inflammatory and oxidant/antioxidant imbalance in the cardiac tissue of Ang II-infused mice improved long-QTc intervals

**Fig 6:**
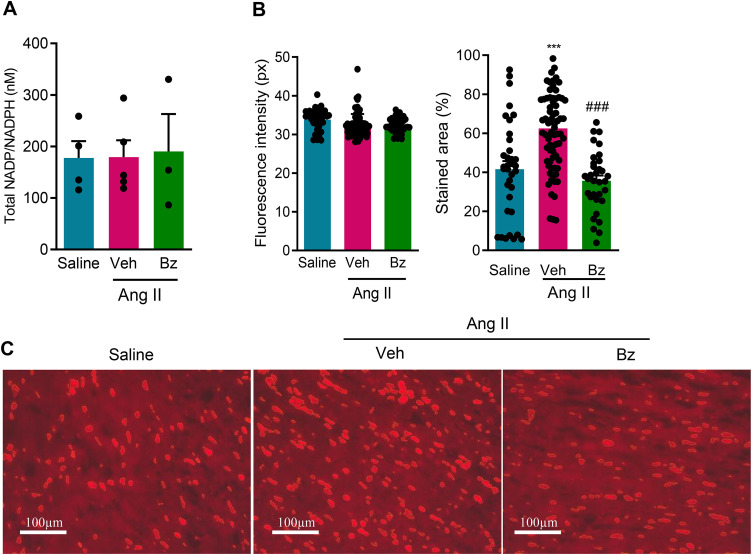
Effect of Bz therapy on ROS production in the cardiac tissue of Ang II-infused mice. (A) A commercial assay kit in ventricle extracts of the studied groups detected concentrations of NADP/NADPH. (B) Graphs show fluorescence intensity (pixel) and area marked by the DHE probe in cardiac tissue. (C) Representative sections of ventricles stained with the ROS probe DHE. Data are expressed as means ± SD. ^***^, p < 0.001, Sal *vs* Ang II infusion; ^###^ p < 0.001, Ang II + Veh *vs* Ang II + Bz (test: ANOVA, post-test: Tukey’s multiple comparisons). Bars = 100 µm.

Here, we tested whether the increased QTc interval dispersion in Ang II-induced pressure overload in C57BL/6 mice is associated with the inflammatory profile, increased ROS, and/or redox imbalance. Furthermore, we sought to understand in our model whether Bz therapy could have a beneficial effect by acting on the oxidative pathway and, consequently, improving the electrical profile. Thus, we evaluated the oxidative axis, assessing the potential generation of ROS via the NADP/NADPH system, the production of ROS reflected by DHE labeling, antioxidant defense, and lipid peroxidation. At 28 dps, similar concentrations of NADP/NADPH were detected in tissue extracts, regardless of the stimulus and treatment administered (Fig 6A). Thus, seeking to clarify the mechanistic action of Ang II and Bz therapy in our model, we evaluated oxidative stress by assessing ROS production in cardiac tissue using the DHE probe. Analysis per microscopic field showed that the intensity of DHE fluorescence was similar in the groups studied (Fig 6B, 6C). However, compared with Sal infusion, Ang II infusion induced an increase in the stained areas of the heart tissue, while Bz therapy reduced the percentage of DHE stained areas, at 28 dps (Fig 6B, 6C). Further, data expressed as analyzed mice per experimental group corroborated that Ang II infusion did not affect the DHE fluorescence intensity (S5 Fig). Again, Bz therapy showed a beneficial effect by reducing the DHE-marked cardiac area compared with the Veh-treated Ang II-infused group (S5 Fig). Interestingly, these data show that 21 days of Bz therapy were sufficient to reduce the DHE-stained area in the heart tissue, compared with Veh administration in Ang II-infused mice, suggesting a possible action of Bz therapy in this axis.

At 28 dps, when the pump capacity of Ang II infusion ended, serum samples were obtained to analyze systemic inflammatory profiles, the heart ventricles were collected, and extracts prepared to study inflammation, oxidative damage, and antioxidant profile. Compared with Sal infusion, Ang II-infused mice exhibited an increase in the concentrations of multiple cytokines in serum, particularly IL-6. In contrast, in the serum of Bz-treated Ang II-infused mice, this tendency was reversed (S6A Fig). Moreover, Ang II infusion tended to increase IL-6 concentrations in cardiac ventricular extracts compared with the Sal-infused group. Again, in ventricle extracts from Ang II-infused mice treated with Bz, IL-6 concentrations tended to be reduced compared with the Veh-infused group (S6B Fig). In the ventricle extracts, the antioxidant process assessed by SOD activity tended to be reduced Ang II-infused mice (Sal, 274.5 ± 31 U/mg normalized per 100 mg tissue *vs* Ang II, 217.6 ± 31 U/mg normalized per 100 mg tissue), while Bz therapy showed similarity to the Sal-infused group (S6C Fig). On the other hand, our analysis of TBARS, as a readout of lipid peroxidation and a downstream biomarker of oxidative stress, revealed increased levels of these products in ventricle extracts of Ang II-infused mice (Sal, 0.156 ± 0.007 OD *vs* Ang II, 0.266 ± 0.553 OD; p < 0.05), while Bz therapy tended to reduce it (S6D Fig). These findings underscore the role of Ang II in oxidative stress, suggesting that Ang II infusion induced an oxidant/antioxidant imbalance.

**Fig 7:**
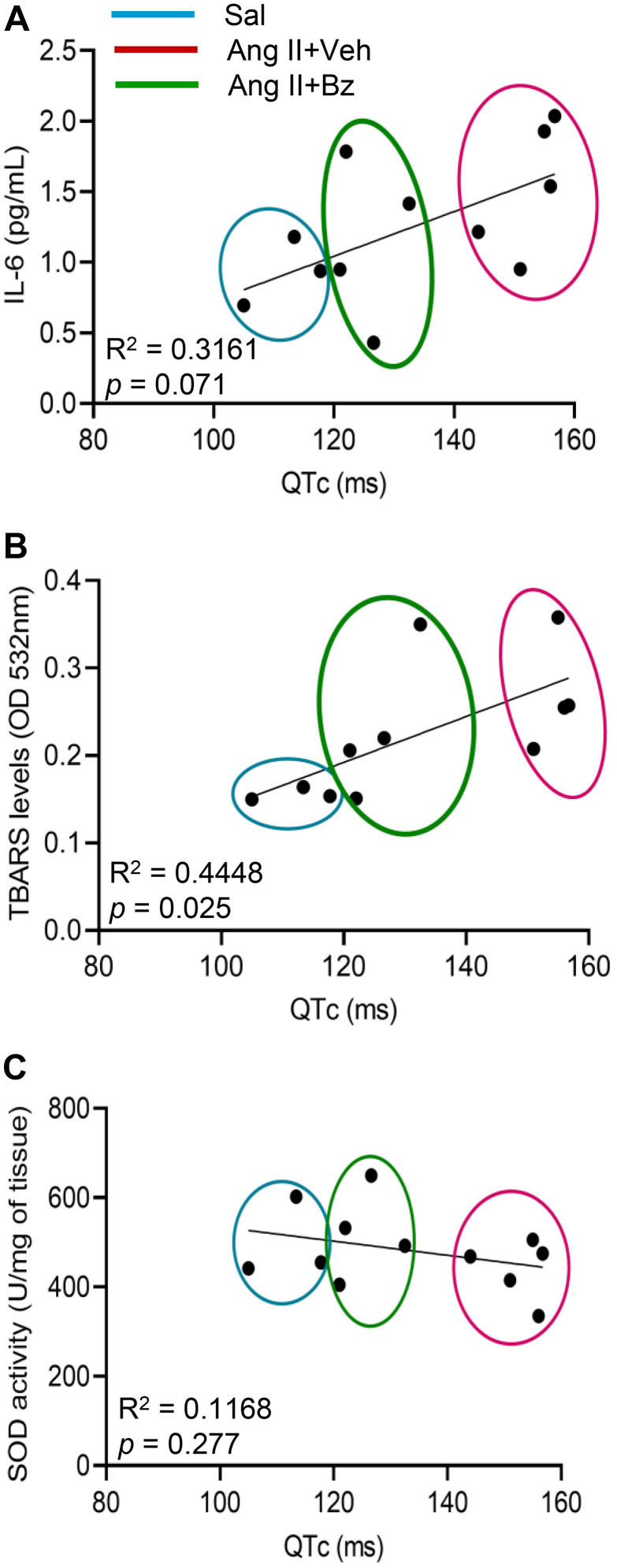
Beneficial effects of Bz on long-QTc were correlated with amelioration of inflammation and antioxidant/oxidant imbalance in cardiac tissue of Ang II-infused mice. (A) The graph shows the correlation of QTc interval dispersion (ms) with IL-6 concentrations (pg/mL) in ventricle extracts. (B) The graph shows the correlation of QTc interval dispersion (ms) with TBARS levels (OD) in ventricle extracts. (C) The graph shows the correlation of QTc interval dispersion (ms) with SOD concentrations (U/mg of tissue) in ventricle extracts. Each dot represents a mouse sample. Ellipses identify groups of mice; color code: blue, saline; magenta, Ang II + Veh; green, Ang II + Bz (Test: Pearson correlation coefficient and two-tailed).

Thus, trying to clarify the relation of these Ang II-induced inflammatory and oxidant/antioxidant alterations in the cardiac tissue of C57BL/6 mice with clinical signs, we evaluated the correlation of these abnormalities with the prolonged QTc interval. First, our analysis showed that the IL-6 concentrations in heart ventricles tend to correlate positively with prolongation of QTc intervals (R^2^ = 0.3161, p = 0.071; Fig 7A). Crucially, TBARS levels in the ventricle extracts correlated directly with the dispersion of QTc intervals (R² = 0.4448, p = 0.025; Fig 7B). In contrast, SOD activity in the heart tissue was not correlated with the long-QTc intervals (R² = 0.1168, p = 0.277; Fig 7C).

**Fig 8:**
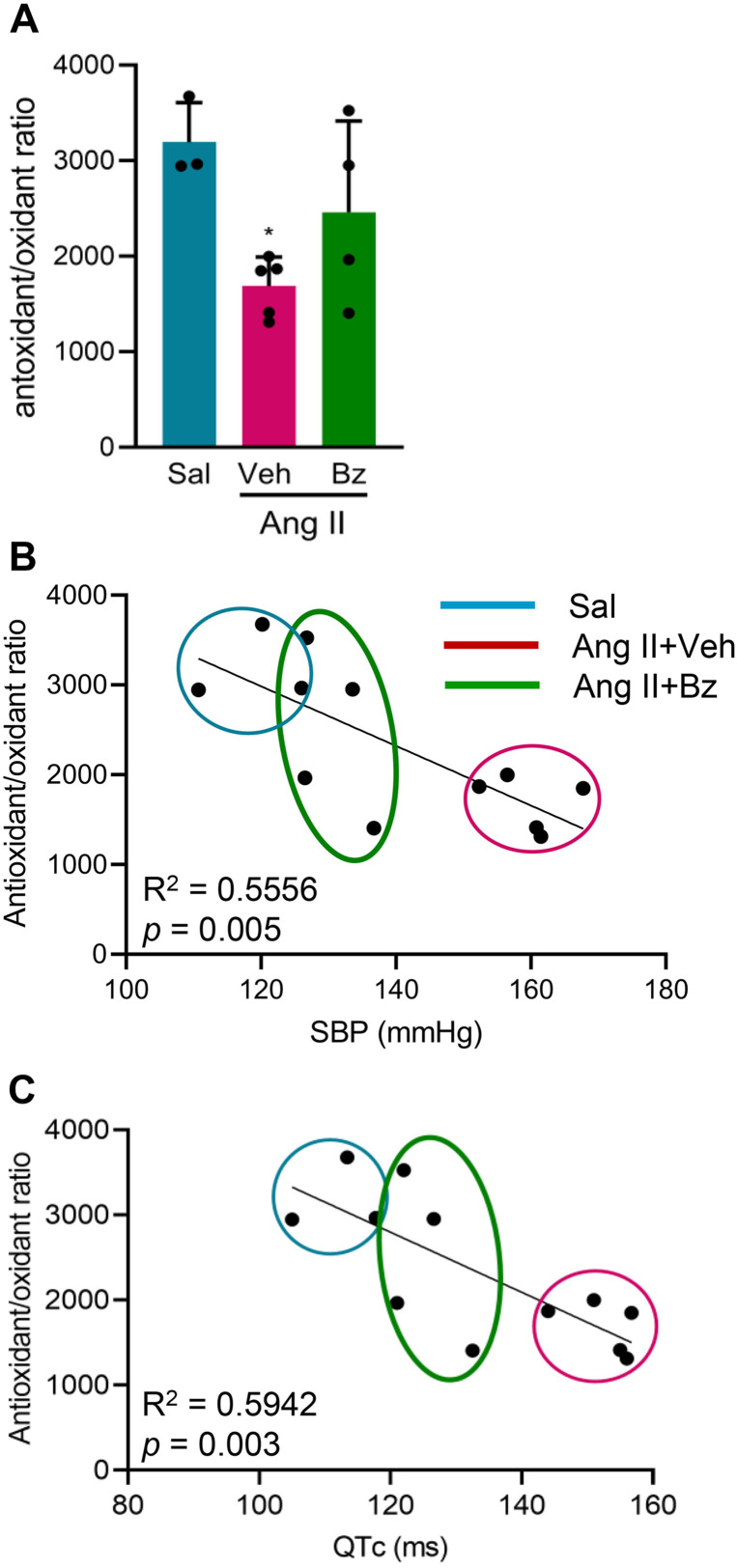
Bz therapy improves pressure overload and QTc interval dispersion by restoring antioxidant/oxidant balance. (A) The graph shows the antioxidant/oxidant ratio (ratio = SOD activity/ TBARS levels) expressed as fold change. (B) The graph shows the correlation of QTc interval prolongation (ms) with SBP (mmHg). (C) The graph shows the correlation of QTc interval dispersion (ms) with the antioxidant/oxidant ratio. Each dot represents a mouse sample. Ellipses identify groups of mice; color code: blue, saline; magenta, Ang II + Veh; green, Ang II + Bz (Test: Pearson correlation coefficient and two-tailed).

Lastly, we analyzed the antioxidant/oxidant ratio in the heart tissue of each mouse, revealing a reduction in this ratio in Veh-treated Ang II-infused mice compared with Sal-infused mice (Sal, 3,195 ± 414-fold change *vs* Ang II + Veh, 1,688 ± 304-fold change; p < 0.05). However, we found a trend towards restoring this balance in heart ventricles was found in Bz-treated mice, suggesting the potential beneficial effect of Bz therapy in our clinical analyses (Fig 8A). The correlation test further revealed a direct correlation between the decrease in antioxidant/oxidant ratios with the systolic pressure overload (and R^2^ = 0.5556, p = 0.005; Fig 8B) and prolonged dispersion of QTc intervals (R^2^ = 0.5942, p = 0.003; Fig 8C). In conclusion, these data underscore the promising effects of Bz therapy on blood pressure and Ang II-induced prolonged QTc, which are associated with reduced inflammation and improved antioxidant/oxidant ratio in cardiac tissue, sparking further interest in its future implications.

## Discussion

In this study, we initially established a model of arterial hypertension induced by Ang II infusion using a 4-week duration minipump implantation in C57BL/6 mice [13], which allowed us to study the effects of Ang II on cardiac electrical conduction. Moreover, this model allowed us to test the putative antioxidant effects of early Bz therapy on increased blood pressure and electrical changes promoted by chronic and continuous Ang II infusion.

To challenge our working hypotheses, we used a preclinical model of Ang II-promoted pressure overload in C57BL/6 mice, with preserved LVEF% and FAC%, as well as in LV and RV areas, and LVID, therefore with preserved cardiac function and structure. Preservation of LVEF was also found in a model of atrial fibrillation in Ang II-infused C57BL/6 mice [33]. Therefore, we established a model of hypertension induced by chronic and continuous Ang II infusion, allowing us to challenge our ideas of early therapeutic strategy. In this model of continuous infusion of Ang II in C57BL/6 mice for 28 days, we observed a body weight loss of 2–3% and a survival rate of 100%, data that we can confidently rely on and corroborate previous findings [34]. Physiologically, Ang II contributes to BP homeostasis. However, chronically augmented levels of Ang II promote hemodynamic imbalance, which contributes to blood pressure overload, inducing hypertension [35,36]. We used a model of Ang II infusion via osmotic minipump implant with an early onset (3 days of infusion) of BP overload [13]. Here, we showed that in Ang II-infused mice, the blood pressure overload resulted in increased MBP, SBP, and DBP at 7, 14, 21, and 28 dps. Further, considering that ARBs act by blocking Ang II receptors, specifically the AT_1_R, critical for increasing BP [37], we used Los therapy as a control, while challenging our ideas. As expected, Los reduced Ang-II-induced BP overload, supporting that this model is promptly responsive to therapeutic intervention. Ang II and AT_1_R have been identified in cells and cardiac muscle and may be involved in increasing cardiac action potential duration [38]. More recently, inflammation has been proposed as an underpinning factor for electrical alterations, particularly the prolongation of QTc intervals, a risk factor for adverse events in infectious and non-infectious cardiovascular diseases [12,39]. Considering the inflammatory nature of Ang II-induced BP overload in C57BL/6 mice [13,14], we tested the effects of Ang II infusion in C57BL/6 mice as a trigger for changes in cardiac electrical physiology. Our study has uncovered a significant implication: the chronic and continuous infusion of Ang II affects cardiac electrical conduction as evidenced by increase in the QRS complex, PR and QTc intervals, supporting prolongation of ventricular repolarization. This finding has the potential to significantly impact our understanding and management of cardiovascular pathologies and risk factors including chronic arterial hypertension and cardiac fibrosis [40,41]. Indeed, inflammation-associated fibrosis has been described in this model of Ang II-induced BP overload in C57BL/6 mice [13]. Changes in heart rate, QTc, and RR intervals are indicators of cardiovascular diseases, mainly underpinned by inflammatory processes [12]. As we have predicted, electrical abnormalities, exceptionally prolonged QTc intervals, were detected in Ang II-infused mice. Crucially, these alterations of heart electrophysiology were dependent on persistently increased levels of Ang II, as the infusion of this renin-angiotensin-aldosterone system product did not trigger a process capable of self-sustaining 30 days after infusion cessation. Thus, we bring evidence that this model is suitable to test the effect of early Bz therapy on blood pressure overload and electrical changes, particularly the prolonged QTc intervals. In a preclinical model of Chagas cardiomyopathy, daily administration of 25 mg/Kg of Bz for 30 days did not show hepatic or renal toxicity [25]. Here, Bz therapy, initiated when blood pressure overload is settled and prolonged for 21 days, reduced the Ang II-induced MBP at all analyzed timepoints. This result suggests that Bz therapy may be acting on mechanisms related to the activation of the angiotensin converting enzyme (ACE)/Ang II/AT_1_R axis, as it is a critical pathway regulating vascular tone and BP homeostasis [4]. It is the first time that the beneficial effect of Bz on BP has been shown. Therefore, it is an exciting result, considering that most of the available medications act as ACE inhibitors or angiotensin receptor antagonists [42]. Thus, our findings opened an intriguing challenge to understand the biological and molecular mechanisms of Bz acting as an antihypertensive drug. Further, the short regimen (21 days) of Bz therapy had borderline beneficial effects on the Ang II-induced increase in RR intervals and QRS complex. RR intervals preserved regular patterns without variations, independently of the infusion (Sal *vs* Ang II) and the therapy administered (Veh *vs* Bz). On the other hand, the duration of the RR intervals was increased in Veh-treated Ang II-infused mice, contrasting with the RR interval duration seen in Bz-treated Ang II-infused mice, that resembles the findings in Sal-infused mice. The increased duration of the RR intervals reflects the change in heart rhythm. It is an indicator of dysregulation of the autonomic nervous system, which is a risk factor for adverse cardiovascular events such as arterial hypertension [43]. In addition to the increased duration of the RR interval, arterial hypertension can compromise cardiac electrical conduction, promoting prolongation of the QRS complex [44]. The partial effect of Bz on the Ang II-induced increase in the QRS complex is indicative of the beneficial effect of this therapy in reducing pressure overload, since the increase in the heart’s workload promoted by hemodynamic changes impacts ventricular depolarization, and consequently the QRS complex [45]. Thus, the effects of Bz therapy on RR interval and QRS complex duration suggest that Bz may have potential therapeutic use in the early phase of cardiovascular diseases associated with chronic arterial hypertension. Our initial hypothesis was focused on the potential modulatory action of Bz on the Ang II-triggered prolonged QTc intervals. Bz therapy had a precocious beneficial effect, reducing the dispersion of the QTc interval after 7 and 14 days of treatment. Nevertheless, the biological mechanisms underlying the therapeutic action of Bz remain unsolved.

Elevated concentrations and chronic release of Ang II induce blood pressure overload, which may be related to the onset of cardiovascular diseases through inflammatory processes and/or oxidative stress [46]. Blood pressure overload can promote an imbalance between the release of ROS and antioxidant defense, changes in cardiac electrophysiology, and other biological consequences such as cardiac fibrosis [47]. Further, the increase in afterload due to chronic elevation of BP can promote adaptive cardiac remodeling, leading to increased heart weight [48]. In our model, the increase of the relative weight of the heart (28 dps) of Ang II-infused C57BL/6 mice may be related to an adaptive process of the heart, due to the increase in afterload derived from pressure overload, as previously seen in a model of Ang II-induced ventricular remodeling [49]. The short regimen of Bz therapy did not interfere with this parameter; however, the Ang II-induced blood pressure overload was controlled after 7 days of Bz administration, i.e., 14 days after initiation of the Ang II infusion, suggesting that this therapy would be ideal for the initial phases of heart remodeling, controlling blood pressure and electrical activity.

Inflammatory activation can influence the electrophysiology of cardiomyocytes through different mechanisms, impacting the duration of the cardiac action potential and, consequently, the ionic flow and the electrical conduction system, contributing to prolongation of the QTc interval [12]. Elevated levels of IL-6 may affect cardiac electrophysiology and are associated with prolongation of the QT interval in cardiovascular diseases [39]. Here, the Ang II infusion induced a trend to elevate the concentrations of IL-6 in the cardiac tissue, which was directly correlated with an increase in QTc interval dispersion. At the same time, Bz therapy tended to reduce it to levels found in cardiac tissue of saline-infused mice. These findings suggest that Bz therapy initiated at 7 days of Ang II infusion, when pressure overload is already settled, may be acting on the inflammatory axis, improving BP and cardiac electrical conduction. It agrees with a model of IL-6-infusion for 7 days, leading to elevated IL-6 concentrations in the cardiac tissue, associated with concentric hypertrophy, a process associated with increased pressure overload and interstitial fibrosis [50]. Bz treatment has immunomodulatory properties down-regulating nitric oxide and cytokine synthesis by LPS- and/or IFN-γ-stimulated murine macrophages [51]. Also, *in vivo,* in LPS-induced hyperactivation in C57BL/6 mice, the immunomodulatory effects of Bz were demonstrated by its ability to increase survival and decrease serum levels of IL-6 and TNF [52]. Furthermore, in a model of Ang II-induced arterial hypertension in C57BL/6 mice, increased BP was associated with elevated plasma IL-6 levels. Also, elevated plasma IL-6 levels were detected in Ang II-triggered endothelial dysfunction and pressure overload [53]. In contrast, IL-6-deficient mice presented reduced BP compared with C57BL/6 mice [54], corroborating the importance of IL-6 in BP regulation. Thus, the reduction of IL-6 levels systemically and in cardiac tissue may underlie the beneficial effect of Bz therapy on BP overload and prolonged QTc intervals.

In addition to the high concentration of IL-6, the generation of ROS by NADPH oxidase may be the basis of Ang II-induced hypertension, promoting cardiovascular damage and electrophysiological changes [46,55]. Under physiological conditions, ROS production and antioxidant capacity are balanced; however, under pathological conditions, increased ROS production is not counterbalanced by antioxidant defense. Activation of systemic and tissue renin-angiotensin aldosterone increases vascular ROS production, and one consequence of oxidative stress is tissue damage due to high levels of free radicals, which can cause oxidative deterioration of lipids, such as lipid peroxidation, implicated in vascular pathologies such as hypertension [56]. Our data show that in the context of ROS production, chronic and continuous Ang II infusion, as well as Bz therapy, did not alter the concentrations of NADP/NADPH in ventricle extracts. In a model of endothelial dysfunction, 7 days of Ang II infusion promoted an increase in systolic blood pressure, with an increase in the activity and expression of NAD(P)H oxidase [57]. In our model, NADP/NADPH was evaluated at the experimental endpoint (28 dps), which may explain our results, as ROS production through this pathway may have occurred earlier, a matter to be clarified. Interestingly, in Ang II-infused mice, an increase in DHE-labelled areas was detected in cardiac tissue. Previous data showed that Ang II infusion induced ROS production in the central nervous system of C57BL/6 mice, as indicated by increased DHE staining [58]. Crucially, our data show that Bz therapy reduced the DHE-stained areas in the cardiac tissue of Ang II-infused mice. In the heart tissue, ROS plays a fundamental role in cellular homeostasis, regulating multiple physiological signaling pathways and biological processes. However, in high concentrations, together with impaired antioxidant activity, ROS can impact heart physiology, promoting arrhythmia [59]. Thus, Bz therapy may be acting on this oxidative axis, controlling ROS generation, an effect that may explain the improvement of clinical and electrical traits, mainly reducing BP overload and the dispersion of the QTc interval.

Increased ROS production and unbalanced antioxidant defense are associated with comorbidities such as hypertension [60]. In our model, continuous high concentrations of Ang II tended to decrease SOD activity in ventricular extracts. Crucially, Bz therapy tended to restore this antioxidant enzyme in patterns similar to those of the saline group. This finding is indicative of the redox imbalance promoted by Ang II infusion and suggests a beneficial effect of Bz therapy on antioxidant mechanisms. In a model of vascular dysfunction induced by Ang II infusion and a diet high in salt intake, a decrease in antioxidant enzymes such as SOD was observed in the brain [61]. Previously, in an experimental model of sepsis, the effect of Bz therapy on this axis was seen as increased activity of glutathione peroxidase and SOD in the liver, and activation of the transcription factor Nrf2 *in vitro* [27].

We next assessed lipid peroxidation, a terminal process of oxidative stress. Ang II infusion promoted lipid peroxidation, reflected by increased TBARS levels in the heart ventricles, and Bz therapy tended to reduce this oxidative damage. Interestingly, TBARS levels correlated with the increase in QTc interval dispersion. We have previously shown the effects of Bz reducing TBARS levels in the central nervous system of chronically *T. cruzi*-infected C57BL/6 mice [24]. However, in this model, one could not rule out an indirect effect of Bz controlling the parasite and, therefore, reducing the stimulus for oxidative stress. Our results were corroborated in an acute *T. cruzi* infection model showing that short treatment with 100 mg/Kg/day of Bz reduced TBARS levels in the brain cortex [62].

Here, we demonstrated that Ang II infusion promotes an imbalance between ROS production (seen as an increase in lipid peroxidation) and antioxidant defense (seen as a reduction of SOD activity) correlated with the increase in blood pressure and QTc interval dispersion. At the same time, Bz therapy partially restored the physiology of this axis, beneficially impacting these clinical alterations. Therefore, our data support that the effects of Bz therapy in improving the Ang II-promoted BP overload and prolonged QTc interval may be underpinned by regulation of the oxidant/antioxidant axis.

## Conclusion and limitations

In a model of Ang II infusion-induced pressure overload and impaired cardiac electrical activity, such as increased QTc interval dispersion, but preserved cardiac function (conserved LVEF without progression to heart failure), we tested short-term (21 days) Bz therapy as an early-phase therapeutic intervention. We brought mechanistic insights into its beneficial action. To our knowledge, this is the first proposal of rational use of Bz therapy in a non-infectious cardiomyopathy based on the inflammatory basis for prolonged QTc interval, a predictor of poor prognosis and risk factors such as cardiovascular events and death in cardiovascular diseases [12,39]. Furthermore, we proposed here that Bz may act directly through anti-inflammatory and antioxidant mechanistic pathways, restoring physiological patterns. Here, we bring evidence that Bz, a low-cost medicament here used here as a short-term and low-dose scheme, had a beneficial effect on pressure overload and electrical changes, particularly on prolonged QTc interval in a model of hypertension induced by Ang II infusion. Further, we provide evidence that Bz therapy has beneficial effects on the inflammatory and oxidant/antioxidant axis, crucial mechanisms underlying the onset and progression of cardiovascular disorders [63]. The results of our study suggest that Bz therapy would be acting mainly on the oxidant/antioxidant axis, restoring the balance between ROS release, and consequently lipid peroxidation, and antioxidant defense, ameliorating clinical signs. Moreover, our data support that Bz therapy deserves further evaluation as an anti-inflammatory and antioxidant adjuvant tool to improve BP overload and long-QTc syndrome underlying cardiovascular diseases. Lastly, the recent synthesis of Bz analogs with no mutagenic potential and low potential for renal and hepatic toxicity [64] opened a perspective of dissociating anti-parasitic, and cardioprotective and immunoregulatory activities of this nitrotriazole medication and the potential of these compounds in the cardiovascular context.

Our work has a crucial limitation, due to the high cost of the preclinical model of Ang II infusion using osmotic minipumps and several clinical and biochemical evaluations, the number of mice per experiment was reduced. Then, to overcome this limitation, we performed three independent experiments, demonstrating the reproducibility of our findings. Another limitation of the present study is the exclusive use of female mice. Several findings show gender differences in the pathophysiology of hypertension, especially the sex hormonal context in the regulation of cardiovascular health [65]. We recognize that including only one gender restricts the generalizability of our findings. Thus, it is essential to conduct complementary studies using male mice, exploring the effects of sex hormones on therapeutic effects of Bz therapy on Ang II-induced hypertension and electrical abnormalities. Moreover, our results deserve to be independently challenged by other groups, using other targets and tools for studying the beneficial effects of Bz, particularly on BP overload and the prolonged QTc intervals.

## Supporting information

S1 Fig Flow chart showing the experimental protocol with the number of animals used. (TIF)

S2 Fig Effect of osmotic minipump implantation on clinical analyses. **(A)** Graph shows survival Kaplan-Meier curve. **(B)** Graphs show body weight of mice during 28 days after the implantation of the osmotic minipump. **(C)** Graphs show the variation in body weight (%) of mice. ^**^, p < 0.01, comparison of Sal-infused group with other groups (tests: Breslow test; ANOVA with Tukey’s multiple comparisons post-test; and 2-way ANOVA).
(TIF)

S3 Fig 30 days after the osmotic minipump started operating, the increase in QTc interval dispersion was restored. **(A)** Experimental design of the blood pressure overload. induced by Ang II infusion through osmotic minipump implantation lasting 28 days, and with an extension of 30 days after the minipump stops working (58 dps). Graphs showing **(B)** heart rate (bpm), QTc interval (ms), **(C)** relative heart weight (mg/g), and **(D)** LVEF% 30 days after the osmotic minipump stopped operating. test: *t*-Student.
(TIF)

S4 Fig Ang II infusion promoted pressure overload. **(A)** Representative images of the kinetics of blood pressure analyses at all analysis points. **(B)** Graphs showing the effect of Ang II infusion and Bz therapy on systolic and diastolic blood pressure at all time points. Data are shown as means ± SD. ^**^, p < 0.01, comparison of Sal-infused group with other groups; ^###^, p < 0.001, Ang II + Veh *vs* Ang II + Bz (tests: *t*-Student, ANOVA with Tukey’s multiple comparisons post-test).
(TIF)

S5 Fig Bz therapy reduces the area marked with ROS after Ang II infusion. Graphs show the effect of Bz therapy on fluorescence intensity and ROS-marked area using the DHE probe. Data are shown as means ± SD. ^#^, p < 0.05, Ang II + Veh *vs* Ang II + Bz (test: ANOVA, post-test: Tukey’s multiple comparisons).
(TIF)

S6 Fig Bz therapy tends to decrease the concentration of IL-6 and TBARS in the ventricle extract. **(A)** Representative images and graphs from FACS analysis of CBA and concentration of the cytokines studied. **(B)** Effect of Ang II infusion and Bz therapy on IL-6 concentration (pg/mL), SOD activity (U/mg of tissue), and TBARS levels (OD 532 nm) in heart ventricle extracts. ^*^, p < 0.05, Sal-infused *vs* Veh-treated Ang-II infused group (test: ANOVA, post-test: Tukey’s multiple comparisons).
(TIF)

S1 Table Data used to build graphs and figures. (DOCX)
