# Correction: Benznidazole therapy improves pressure overload and cardiac electrical profile in an experimental model of Angiotensin II infusion-induced hypertension: Mechanistic insights

**DOI:** 10.1371/journal.pone.0345848

**Published:** 2026-03-26

**Authors:** The PLOS One Staff

## Notice of Republication

The first author’s name is spelled incorrectly. The correct name is: Ana Paula da Silva Pinheiro.

## Supporting information

S1 File Uncorrected version. (PDF)

S2 File Correct version. (PDF)
