# Supplementary figures and images for: Benznidazole therapy improves pressure overload and cardiac electrical profile in an experimental model of Angiotensin II infusion-induced hypertension: Mechanistic insights
Source: PLoS One. 2026 Jan 27;21(1):e0340280. doi: 10.1371/journal.pone.0340280 (PMC12843581; doi:10.1371/journal.pone.0340280)

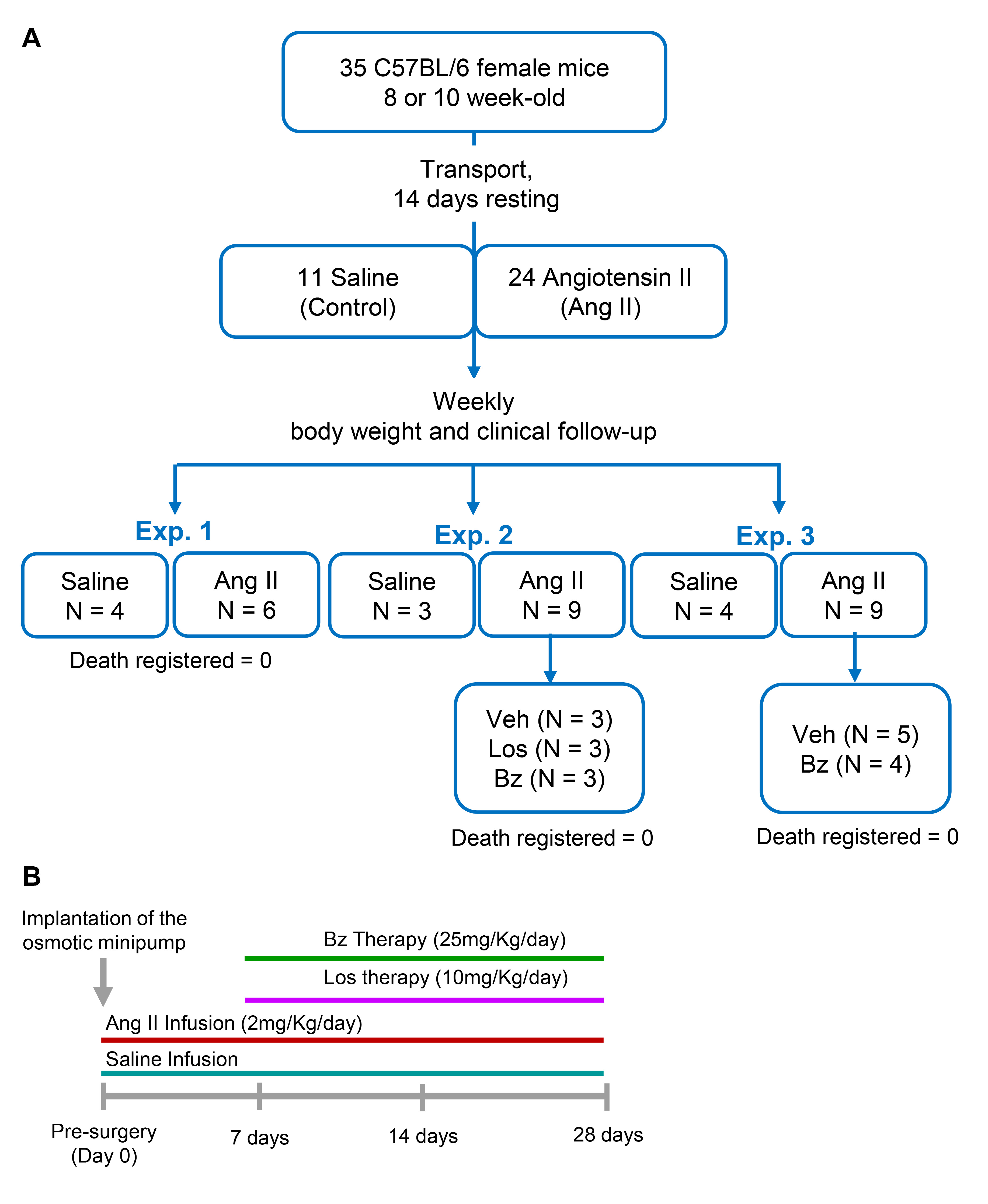

Supplement: S1 Fig — (TIF) [file pone.0340280.s001.tif]

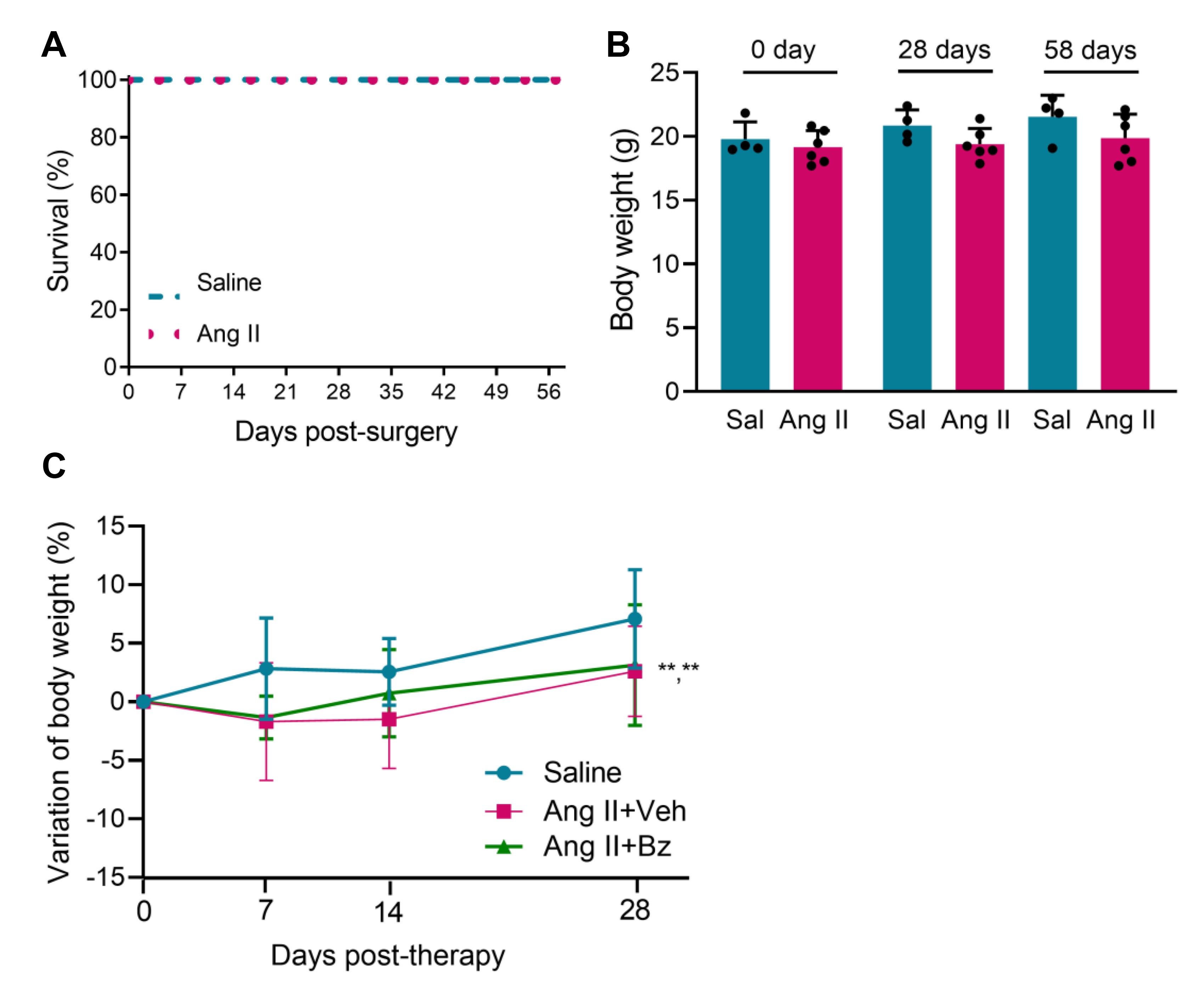

Supplement: S2 Fig — (A) Graph shows survival Kaplan-Meier curve. (B) Graphs show body weight of mice during 28 days after the implantation of the osmotic minipump. (C) Graphs show the variation in body weight (%) of mice. **, p < 0.01, comparison of Sal-infused group with other groups (tests: Breslow test; ANOVA with Tukey’s multiple comparisons post-test; and 2-way ANOVA). (TIF) [file pone.0340280.s002.tif]

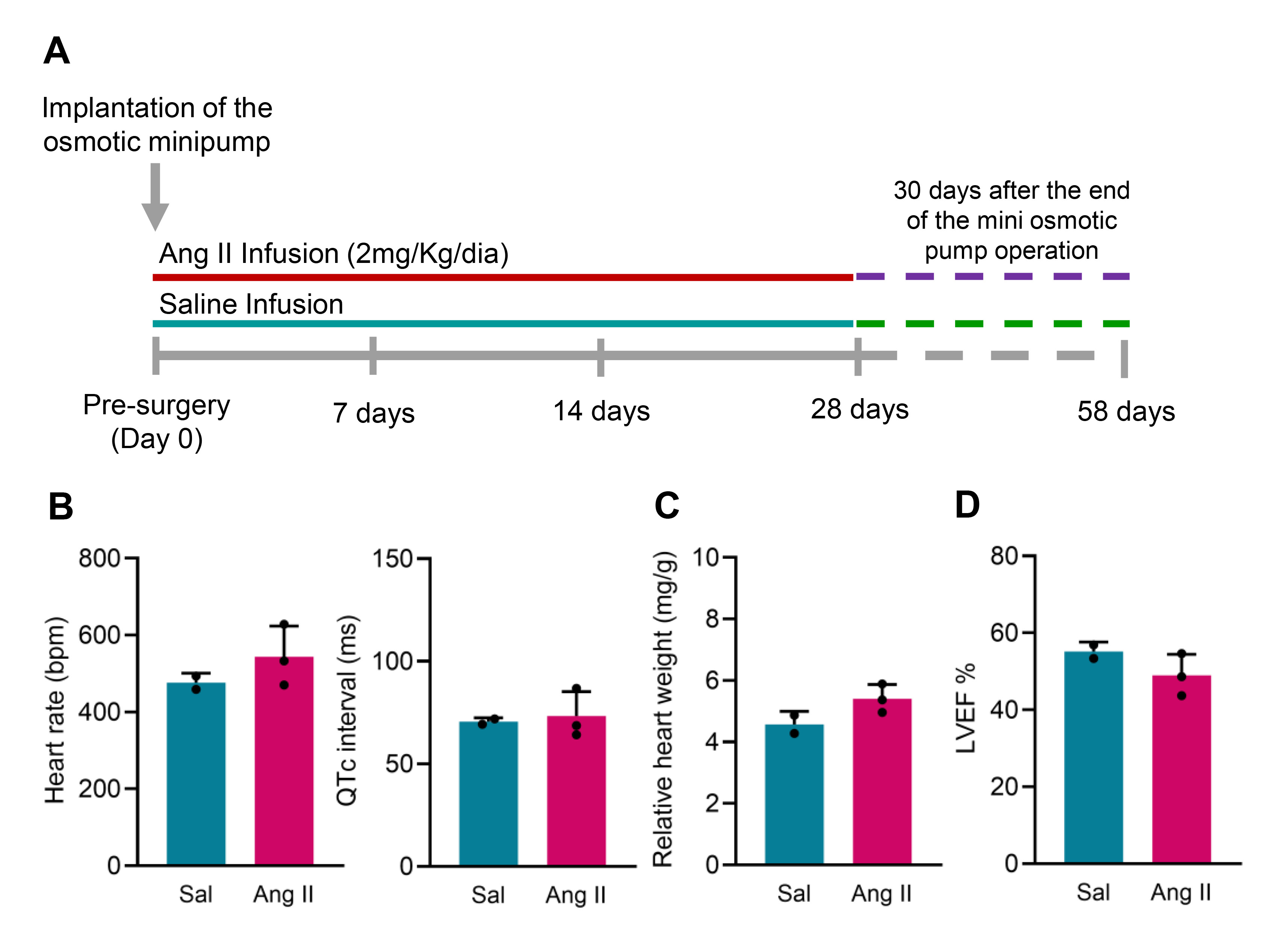

Supplement: S3 Fig — (A) Experimental design of the blood pressure overload. induced by Ang II infusion through osmotic minipump implantation lasting 28 days, and with an extension of 30 days after the minipump stops working (58 dps). Graphs showing (B) heart rate (bpm), QTc interval (ms), (C) relative heart weight (mg/g), and (D) LVEF% 30 days after the osmotic minipump stopped operating. test: t-Student. (TIF) [file pone.0340280.s003.tif]

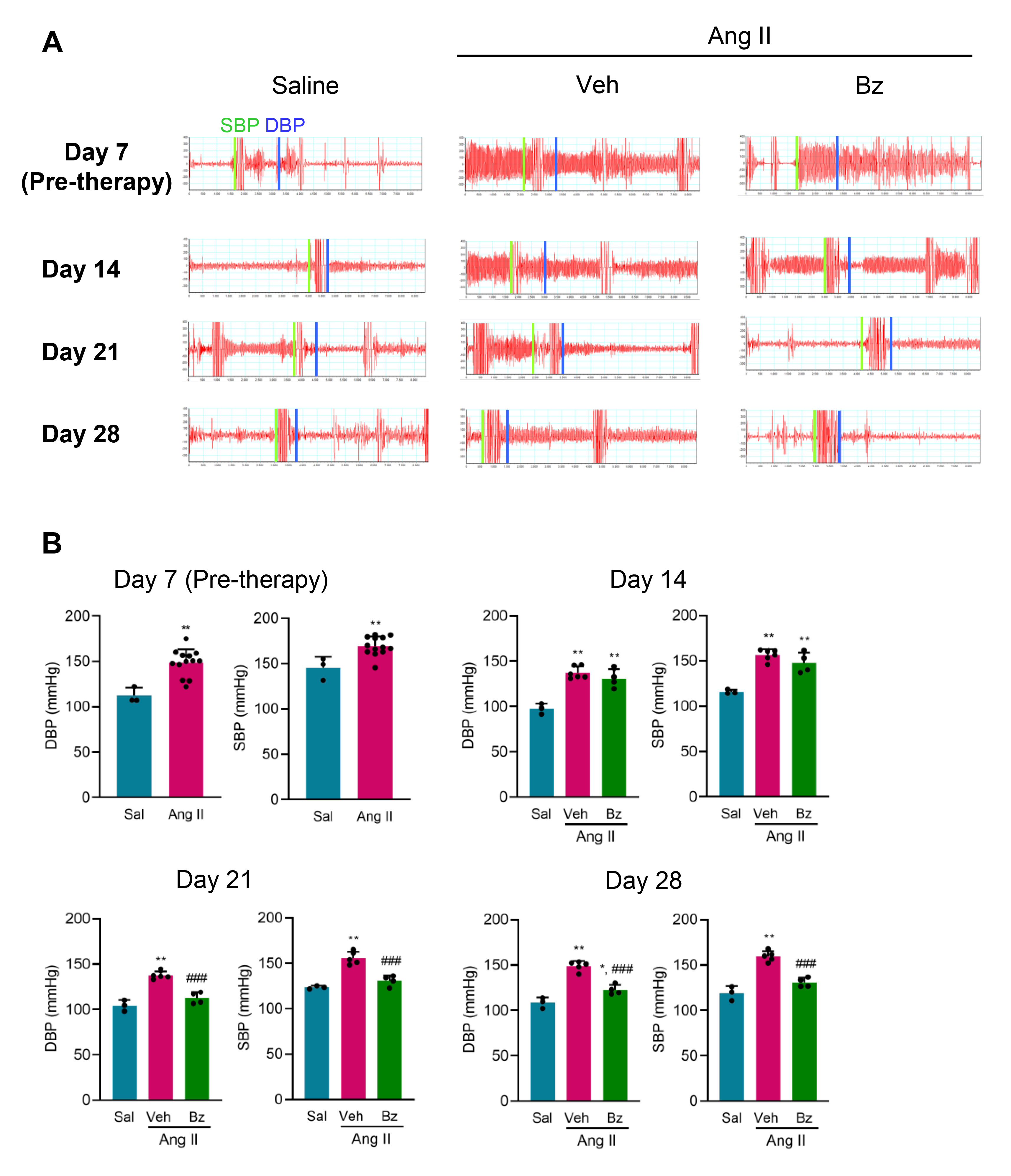

Supplement: S4 Fig — (A) Representative images of the kinetics of blood pressure analyses at all analysis points. (B) Graphs showing the effect of Ang II infusion and Bz therapy on systolic and diastolic blood pressure at all time points. Data are shown as means ± SD. **, p < 0.01, comparison of Sal-infused group with other groups; ###, p < 0.001, Ang II + Veh vs Ang II + Bz (tests: t-Student, ANOVA with Tukey’s multiple comparisons post-test). (TIF) [file pone.0340280.s004.tif]

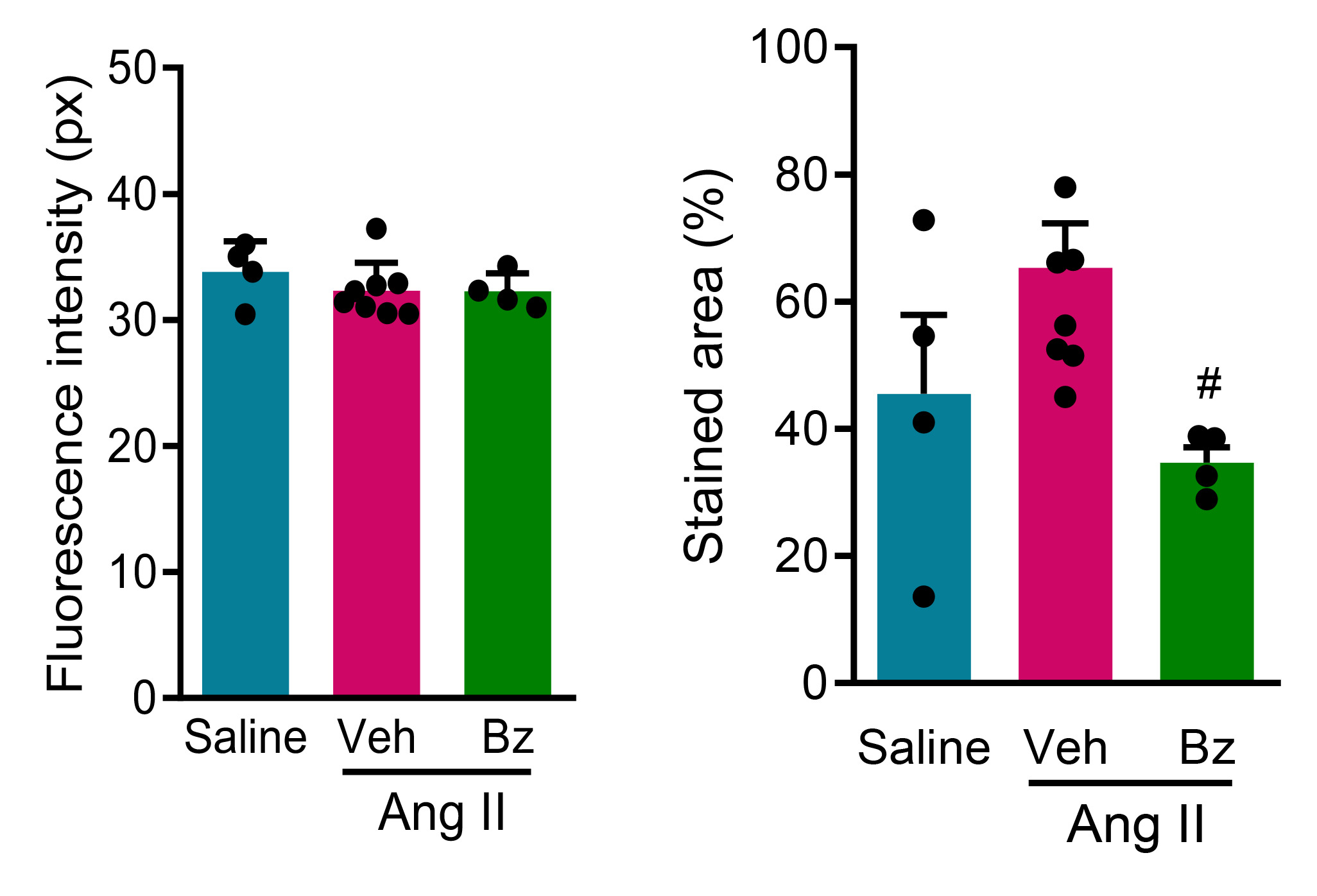

Supplement: S5 Fig — Graphs show the effect of Bz therapy on fluorescence intensity and ROS-marked area using the DHE probe. Data are shown as means ± SD. #, p < 0.05, Ang II + Veh vs Ang II + Bz (test: ANOVA, post-test: Tukey’s multiple comparisons). (TIF) [file pone.0340280.s005.tif]

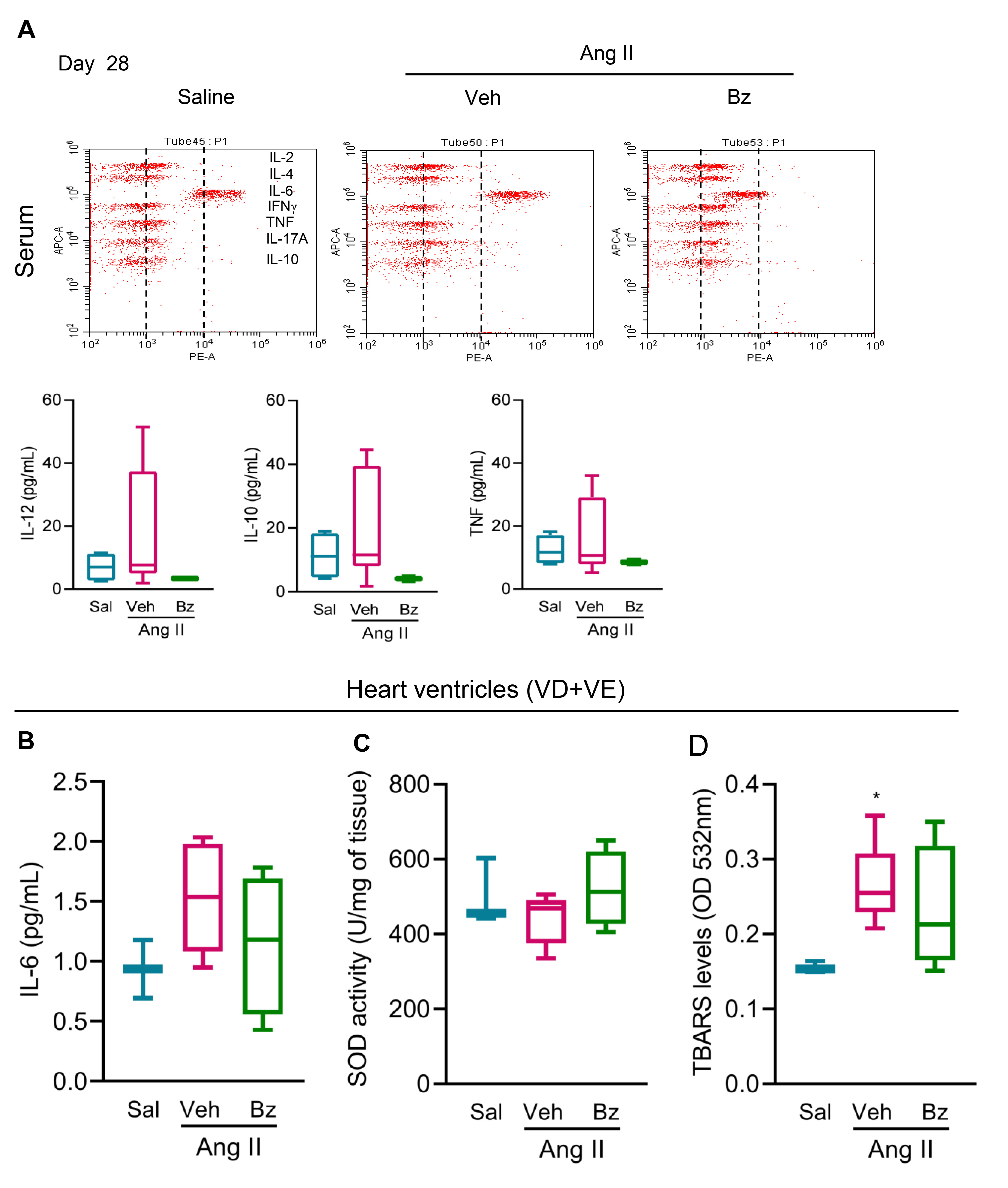

Supplement: S6 Fig — (A) Representative images and graphs from FACS analysis of CBA and concentration of the cytokines studied. (B) Effect of Ang II infusion and Bz therapy on IL-6 concentration (pg/mL), SOD activity (U/mg of tissue), and TBARS levels (OD 532 nm) in heart ventricle extracts. *, p < 0.05, Sal-infused vs Veh-treated Ang-II infused group (test: ANOVA, post-test: Tukey’s multiple comparisons). (TIF) [file pone.0340280.s006.tif]
