# Supplementary material for: Benznidazole therapy improves pressure overload and cardiac electrical profile in an experimental model of Angiotensin II infusion-induced hypertension: Mechanistic insights
Source: PLoS One. 2026 Jan 27;21(1):e0340280. doi: 10.1371/journal.pone.0340280 (PMC12843581; doi:10.1371/journal.pone.0340280)
Supplement: S1 Table — (DOCX) [file pone.0340280.s007.docx]

**PONE-D-25-44084: Benznidazole therapy improves pressure overload and cardiac electrical profile in an experimental model of angiotensin II infusion-induced hypertension: mechanistic insights. Pinheiro et al.**

**S1 Table.** Data used to build graphs and figures.

| **Fig 1A** | Representative images of blood flow at 7-days post-surgery. |
| --- | --- |

| **Fig 1B** | Systolic blood pressure (mmHg) | | | | | | | |
| --- | --- | --- | --- | --- | --- | --- | --- | --- |
| **Days post-surgery** | **7** | | **14** | | | **28** | | |
|  | Sal | Ang II | Sal | Ang II+Veh | Ang II+Los | Sal | Ang II+Veh | Ang II+Los |
|  | 100 | 149 | 128 | 174 | 139 | 117.75 | 161 | 123 |
|  | 108 | 143 | 124 | 179 | 137 | 105 | 168 | 125 |
|  | 107 | 172 | 126 | 177 | 141 | 113.4 | 171 | 127 |
| Mean ± SD | 105 ± 4.36 | 154.67 ± 15.31 | 126 ± 2 | 176.67 ± 2.52 | 139 ± 2 | 112.05 ± 6.48 | 166.67 ± 5.13 | 125 ± 2 |
|  |  | ***. p < 0.001 |  | ***. p < 0.001 | ###. p< 0.001 |  | ***. p < 0.001 | ###. p< 0.001 |

| **Fig 1C** | Representative images of the ventricular chamber | | | |
| --- | --- | --- | --- | --- |
|  | | | | |
| **28 Days post-surgery** | **FAC %** | | **LVEF %** | |
|  | Sal | Ang II | Sal | Ang II |
|  | 52.27 | 51.35 | 54.88 | 49.62 |
|  | 40.32 | 56.66 | 52.57 | 60.98 |
|  | 67.92 | 29.99 | 71.83 | 55.11 |
|  | 63.53 | 42.61 | 62.18 | 63.75 |
|  | 54.62 | 63.32 | 65.25 | 67.62 |
|  | 60.92 | 51.44 | 58.09 | 61.41 |
|  | 19.04 | 59.5 | 53.12 | 65.42 |
|  | 41.1 | 59.12 | 62.37 | 58.3 |
|  |  | 56.28 |  | 62.81 |
|  |  | 61.1 |  | 67.79 |
| Mean ± SD | 49.96 ± 15.95 | 53.14 ± 10.10 | 60.03 ± 6.66 | 61.28 ± 5.69 |
|  |  | p > 0.05 |  | p > 0.05 |

| **Fig 1D** | Representative images of the 3D ECG tracing recorded at 14- and 28-days post-surgery. |
| --- | --- |

| **Fig 1E** | ECG evaluation at 28-days post-surgery | | | | | |
| --- | --- | --- | --- | --- | --- | --- |
| **28 Days post-surgery** | **Heart rate (bpm)** | | **PR interval (ms)** | | **QTc interval (ms)** | |
|  | Sal | Ang II | Sal | Ang II | Sal | Ang II |
|  | 590 | 487 | 36.02 | 35.16 | 63.43 | 82.58 |
|  | 565.8 | 448 | 36.75 | 42.44 | 80.45 | 90.2 |
|  | 521.4 | 471.5 | 34.92 | 48.01 | 60.14 | 66.59 |
|  | 468.4 | 571.2 | 36.27 | 40.13 | 68.47 | 88.69 |
|  | 696.9 | 534.6 | 35.51 | 43.54 | 68.13 | 80.1 |
|  | 657.9 | 448 | 36.56 | 46.51 | 78.03 | 73.86 |
|  | 505.1 | 467.7 | 41.52 | 42.84 | 65.98 | 136.9 |
|  | 504.8 | 553 | 39.3 | 37.85 | 75.5 | 103.6 |
|  | 590.4 | 476.4 | 38.7 | 46.72 | 72.3 | 103.9 |
|  | 514.4 | 495.5 | 40.8 | 40.9 | 70.5 | 88.7 |
|  |  | 482.9 |  | 41.1 |  | 91.5 |
|  |  | 433.5 |  | 41.1 |  | 98.8 |
| Mean ± SD | 561.51 ± 73.17 | 489.11 ± 43.04 | 37.63 ± 2.29 | 42.19 ± 3.72 | 70.29 ± 6.42 | 92.12 ± 18.01 |
|  |  | **. p< 0.01 |  | **. p< 0.01 |  | ***. p< 0.001 |

| **Fig 1F** | ECG evaluation | |
| --- | --- | --- |
| **58 Days post-surgery** | **QTc interval (ms)** | |
|  | Sal | Ang II |
|  | 71.93 | 86.84 |
|  | 69.29 | 68.72 |
|  |  | 64.19 |
| Mean ± SD | 70.61 ± 1.87 | 73.25 ± 11.99 |
|  |  | p > 0.05 |

| **Fig 2A** | Mean blood pressure (mmHg) | | | | | | | |
| --- | --- | --- | --- | --- | --- | --- | --- | --- |
| **Days post-surgery** | **7** | | **14** | | | **28** | | |
|  | Sal | Ang II | Sal | Ang II+Veh | Ang II+Bz | Sal | Ang II+Veh | Ang II+Bz |
|  | 108 | 143 | 128 | 174 | 151 | 113 | 168 | 168 |
|  | 107 | 172 | 124 | 179 | 124 | 127 | 171 | 121 |
|  | 122 | 144.6667 | 107 | 150.5 | 131 | 117.75 | 155 | 122 |
|  | 115 | 148.5 | 100.5 | 146 | 128.3 | 105 | 151 | 126.6 |
|  | 133.4 | 155.7 | 104.2 | 152.4 | 136 | 113.4 | 156.7 | 132.5 |
|  |  | 167 |  | 155 |  |  | 156 |  |
|  |  | 159.6667 |  | 143.5 |  |  | 144 |  |
|  |  | 163.5 |  | 139.5 |  |  |  |  |
| Mean ± SD | 117.07 ± 10.92 | 156.75 ± 10.66 | 112.75 ± 12.39 | 154.98 ± 14.23 | 134.06 ± 10.42 | 115.39 ± 8.03 | 157.39 ± 9.36 | 134.02 ± 19.53 |
|  |  | ***. p < 0.001 |  | ***. p < 0.001 | *. p < 0.05  #. p< 0.05 |  | ***. p < 0.001 | *. p < 0.05  #. p< 0.05 |

| **Fig 2B** | Representative images of blood flow at 21- and 28-days post-surgery. |
| --- | --- |

| **Fig 3A** | Heart rate (bpm) | | | | | | |
| --- | --- | --- | --- | --- | --- | --- | --- |
| **Days of experiment** | **0 (pre-surgery)** | **14** | | | **28** | | |
|  | All mice | Sal | Ang II+Veh | Ang II+Bz | Sal | Ang II+Veh | Ang II+Bz |
|  | 583.1 | 583.1 | 502.1 | 504 | 696.9 | 467.7 | 557.6 |
|  | 627.6 | 678 | 585.4 | 408.2 | 657.9 | 553 | 603 |
|  | 474.7 | 471 | 487 | 534 | 505.1 | 476.4 | 607.6 |
|  | 649 | 509.8 | 499.5 | 531.5 | 504.8 | 499.5 | 418.7 |
|  | 678.2 | 623.1 | 465.8 | 495.7 | 590.4 | 465.8 | 571.9 |
|  | 659 | 593.8 | 474 | 583.1 | 514.4 | 474 | 494.9 |
|  | 620.5 |  | 450.9 | 570.2 |  | 467.9 |  |
|  | 583.7 |  | 453.4 |  |  | 413.4 |  |
|  | 664.5 |  | 495.9 |  |  |  |  |
|  | 585.4 |  |  |  |  |  |  |
|  | 563.4 |  |  |  |  |  |  |
|  | 574.3 |  |  |  |  |  |  |
|  | 670.3 |  |  |  |  |  |  |
|  | 601.3 |  |  |  |  |  |  |
|  | 669.3 |  |  |  |  |  |  |
|  | 615.1 |  |  |  |  |  |  |
|  | 523.1 |  |  |  |  |  |  |
|  | 670 |  |  |  |  |  |  |
|  | 491.5 |  |  |  |  |  |  |
|  | 539.7 |  |  |  |  |  |  |
|  | 525.3 |  |  |  |  |  |  |
|  | 566.3 |  |  |  |  |  |  |
|  | 512.9 |  |  |  |  |  |  |
|  | 621.4 |  |  |  |  |  |  |
|  | 628.8 |  |  |  |  |  |  |
|  | 658.1 |  |  |  |  |  |  |
| Mean ± SD | 598.33 ± 60.30 | 576.47 ± 75.36 | 490.45 ± 40.45 | 518.10 ± 57.95 | 578.25 ± 84.13 | 477.21 ± 38.97 | 542.28 ± 72.91 |
|  |  |  | &&&. p < 0.001  *. p < 0.05 | p > 0.05 |  | &&&. p < 0.001  *. p < 0.05 | p > 0.05 |

| **Fig 3B** | Representative images of the 2D and 3D ECG tracing recorded at 14- and 28-days post-surgery. |
| --- | --- |

| **Fig 4A** | Representative images of the 2D ECG tracing recorded at 28-days post-surgery. |
| --- | --- |

| **Fig 4B** | Representative profiles of RR interval duration (ms) registered in sequential heartbeats. at 28-days post-surgery. |
| --- | --- |

| **Fig 4C** | QRS complex (ms) | | | | | | |
| --- | --- | --- | --- | --- | --- | --- | --- |
| **Days of experiment** | **0 (pre-surgery)** | **14** | | | **28** | | |
|  | All mice | Sal | Ang II+Veh | Ang II+Bz | Sal | Ang II+Veh | Ang II+Bz |
|  | 10 | 10.05 | 13.89 | 13 | 10 | 13.65 | 12.02 |
|  | 11 | 11.67 | 12.59 | 12 | 11.41 | 12 | 12 |
|  | 10.19 | 11.98 | 13.48 | 11 | 10 | 14.19 | 12.1 |
|  | 10 |  |  |  |  |  |  |
|  | 11 |  |  |  |  |  |  |
|  | 12 |  |  |  |  |  |  |
|  | 11 |  |  |  |  |  |  |
|  | 10 |  |  |  |  |  |  |
|  | 11 |  |  |  |  |  |  |
|  | 10 |  |  |  |  |  |  |
|  | 11 |  |  |  |  |  |  |
|  | 13.7 |  |  |  |  |  |  |
| Mean ± SD | 10.91 ± 1.08 | 11.23 ± 1.04 | 13.32 ± 0.66 | 12.00 ± 1.00 | 10.47 ± 0.81 | 13.28 ± 1.14 | 12.04 ± 0.05 |
|  |  |  | &. p < 0.05 | p > 0.05 |  | &. p < 0.05  *. p < 0.05 | p > 0.05 |

| **Fig 4D** | QTc interval (ms) | | | | | | |
| --- | --- | --- | --- | --- | --- | --- | --- |
| **Days of experiment** | **0 (pre-surgery)** | **14** | | | **28** | | |
|  | All mice | Sal | Ang II+Veh | Ang II+Bz | Sal | Ang II+Veh | Ang II+Bz |
|  | 71.27 | 77.72 | 109.3 | 59.61 | 68.13 | 136.9 | 90.98 |
|  | 69.48 | 75.46 | 102.3 | 87.49 | 78.03 | 103.6 | 76.36 |
|  | 74.36 | 72.53 | 107.6 | 91.6 | 65.98 | 103.9 | 83.5 |
|  | 63.44 |  |  |  |  |  |  |
|  | 62.11 |  |  |  |  |  |  |
|  | 58.96 |  |  |  |  |  |  |
|  | 64.72 |  |  |  |  |  |  |
|  | 69.4 |  |  |  |  |  |  |
|  | 67.87 |  |  |  |  |  |  |
|  | 68.88 |  |  |  |  |  |  |
|  | 66.86 |  |  |  |  |  |  |
|  | 70.24 |  |  |  |  |  |  |
| Mean ± SD | 67.30 ± 4.31 | 75.24 ± 2.60 | 106.40 ± 3.65 | 79.57 ± 14.40 | 70.10 ± 6.43 | 114.80 ± 19.14 | 86.61 ± 7.31 |
|  |  |  | &&&. p < 0.001  ***. p < 0.001 | p > 0.05 |  | &&&. p < 0.001  ***. p < 0.001 | #. p< 0.05 |

| **Fig 5A** | Representative images of hearts. showing their dimensions (**a**. **b.** and **c** axis) at 28-days post-surgery. |
| --- | --- |

| **Fig 5A** | Heart dimensions | | | | | |
| --- | --- | --- | --- | --- | --- | --- |
| **28 Days post-surgery** | **Axis A (cm)** | | **Axis B (cm)** | | **Axis C (cm)** | |
|  | Sal | Ang II | Sal | Ang II | Sal | Ang II |
|  | 0.59 | 0.685 | 0.415 | 0.525 | 0.38 | 0.425 |
|  | 0.615 | 0.725 | 0.405 | 0.57 | 0.385 | 0.445 |
|  | 0.695 | 0.665 | 0.46 | 0.52 | 0.41 | 0.43 |
|  |  | 0.835 |  | 0.605 |  | 0.505 |
|  |  | 0.615 |  | 0.51 |  | 0.415 |
| Mean ± SD | 0.63 ± 0.05 | 0.71 ± 0.08 | 0.43 ± 0.03 | 0.55 ± 0.04 | 0.39 ± 0.02 | 0.44 ± 0.04 |
|  |  | p > 0.05 |  | **. p< 0.01 |  | p > 0.05 |

| **Fig 5B** | Representative images of the cardiac ventricular chamber visualized by ECHO analysis at 28-days post-surgery. |
| --- | --- |

| **Fig 5B** | Relative heart weight (mg/g) | | |
| --- | --- | --- | --- |
| **28 Days post-surgery** |  |  |  |
|  | Sal | Ang II+Veh | Ang II+Bz |
|  | 6.72 | 7.34 | 6.88 |
|  | 5.36 | 9.60 | 7.33 |
|  | 5.80 | 6.74 | 7.24 |
|  | 5.46 | 6.28 | 6.43 |
|  | 5.34 | 6.99 | 7.56 |
|  | 6.03 | 10.16 | 8.27 |
|  |  | 5.99 |  |
| Mean ± SD | 5.83 ± 0.62 | 7.49 ± 1.47 | 6.97 ± 0.41 |
|  |  | *. p < 0.05 | p > 0.05 |

| **Fig 5C** | Representative images of the cardiac ventricular chamber visualized by ECHO analysis at 28-days post-surgery. |
| --- | --- |

| **Fig 5D** | ECHO evaluation at 28 days post-surgery | | | | | |
| --- | --- | --- | --- | --- | --- | --- |
| **28 Days post-surgery** | **FAC %** | | | **LVEF %** | | |
|  | Sal | Ang II+Veh | Ang II+Bz | Sal | Ang II+Veh | Ang II+Bz |
|  | 52.27 | 51.35 | 54.44 | 54.88 | 49.62 | 55.08 |
|  | 40.32 | 56.66 | 36.81 | 52.57 | 60.98 | 54.67 |
|  | 67.92 | 29.99 | 33.65 | 71.83 | 55.11 | 58.46 |
|  | 63.53 | 42.61 | 57.75 | 62.18 | 63.75 | 52.63 |
|  | 54.62 | 63.32 | 22.78 | 65.25 | 67.62 | 51.05 |
|  | 60.92 | 51.44 | 69.43 | 58.09 | 61.41 | 59.62 |
|  | 19.04 | 59.5 |  | 53.12 | 65.42 |  |
|  | 41.1 | 59.12 |  | 63.37 | 58.3 |  |
|  |  | 56.28 |  |  | 62.81 |  |
|  |  | 61.1 |  |  | 67.79 |  |
| Mean ± SD | 49.97 ± 15.95 | 53.14 ± 10.10 | 45.81 ± 17.52 | 60.16 ± 6.72 | 61.28 ± 5.69 | 55.25 ± 3.29 |
|  |  | p > 0.05 | p > 0.05 |  | p > 0.05 | p > 0.05 |

| **Fig 5E** | ECHO evaluation at 28 days post-surgery | | | | | | | | |
| --- | --- | --- | --- | --- | --- | --- | --- | --- | --- |
| **28 Days post-surgery** | **RV area (mm)** | | | **LV area (mm)** | | | **LVIDd (mm)** | | |
|  | Sal | Ang II+Veh | Ang II+Bz | Sal | Ang II+Veh | Ang II+Bz | Sal | Ang II+Veh | Ang II+Bz |
|  | 15.11 | 12.7 | 9.11 | 13.11 | 7.74 | 7.58 | 3.99 | 3.45 | 3.12 |
|  | 12.81 | 13.33 | 12.13 | 11.93 | 8.24 | 9.25 | 3.75 | 4.02 | 3.8 |
|  | 11.53 | 12.02 | 14.82 | 8.91 | 7.86 | 5.16 | 3.64 | 2.93 | 2.24 |
|  | 9.43 | 10.5 | 11.87 | 7.86 | 9.02 | 7.21 | 3.58 | 2.95 | 2.82 |
|  | 9.85 | 9.82 | 13.01 | 7.31 | 11.22 | 11.87 | 3.69 | 3.66 | 4.07 |
|  | 8.83 | 12.03 | 12.31 | 8.1 | 7.26 | 8.69 | 3.2 | 3.75 | 2.6 |
|  | 14.3 | 13.8 |  | 11.03 | 6.57 |  |  | 2.68 |  |
|  | 14.05 | 11.65 |  | 8.47 | 8.25 |  |  |  |  |
|  |  | 15.76 |  |  | 9.22 |  |  |  |  |
|  |  | 10.86 |  |  | 8.12 |  |  |  |  |
| Mean ± SD | 11.99 ± 2.43 | 12.25 ± 1.75 | 12.21 ± 1.85 | 9.59 ± 2.14 | 8.35 ± 1.27 | 8.29 ± 3.29 | 3.64 ± 0.26 | 3.35 ± 0.50 | 3.11 ± 0.71 |
|  |  | p > 0.05 | p > 0.05 |  | p > 0.05 | p > 0.05 |  | p > 0.05 | p > 0.05 |

| **Fig 6A** | **Total NADP/NADPH (nM)** | | |
| --- | --- | --- | --- |
| **28 Days post-surgery** |  |  |  |
|  | Sal | Ang II+Veh | Ang II+Bz |
|  | 201.0016 | 118.8834 | 330.2216 |
|  | 258.5642 | 131.6754 | 86.43006 |
|  | 135.019 | 144.064 | 153.8993 |
|  | 115.8552 | 293.9075 |  |
|  |  | 208.7897 |  |
| Mean ± SD | 177.61± 65.14 | 179.46 ± 72.74 | 190.18 ± 125.88 |
|  |  | p > 0.05 | p > 0.05 |

| **Fig 6B** | **Fluorescence intensity (px)** | | |
| --- | --- | --- | --- |
| **28 Days post-surgery** |  |  |  |
|  | Sal | Ang II+Veh | Ang II+Bz |
|  | 28.64 | 34.51 | 31.09 |
|  | 28.49 | 36.17 | 31.77 |
|  | 29.29 | 32.83 | 31.09 |
|  | 28.6 | 31.31 | 30.1 |
|  | 29.93 | 29.95 | 31.04 |
|  | 30.36 | 32.52 | 31.97 |
|  | 32.43 | 32 | 29.91 |
|  | 33.06 | 29.94 | 33.11 |
|  | 30.61 | 31.38 | 28.97 |
|  | 32.99 | 31.54 | 31.49 |
|  | 34.7 | 32.79 | 31.37 |
|  | 34.33 | 32.08 | 34.76 |
|  | 34.83 | 32.16 | 34.69 |
|  | 35.78 | 29.67 | 31.93 |
|  | 33.93 | 30.25 | 33.43 |
|  | 34.91 | 32.12 | 31.92 |
|  | 33.96 | 30.35 | 29.55 |
|  | 35.95 | 31.71 | 31.78 |
|  | 34.89 | 31.74 | 32.2 |
|  | 37.04 | 31.48 | 31.84 |
|  | 33.59 | 30.25 | 32.81 |
|  | 31.25 | 29.57 | 28.82 |
|  | 36.12 | 30.64 | 34.32 |
|  | 32.16 | 32.49 | 34.57 |
|  | 33.12 | 29.5 | 32.17 |
|  | 33.16 | 30.09 | 31.3 |
|  | 34.7 | 31.45 | 29.83 |
|  | 30.08 | 28.83 | 31.52 |
|  | 37.35 | 29.61 | 28.9 |
|  | 35.33 | 30.47 | 33.55 |
|  | 35.32 | 29.57 | 33.41 |
|  | 34.67 | 32.67 | 35.64 |
|  | 35.98 | 29.6 | 33.54 |
|  | 35.05 | 29.03 | 33.08 |
|  | 35.05 | 32.71 | 34.2 |
|  | 35.15 | 33.57 | 33.91 |
|  | 36.92 | 28.2 | 33.85 |
|  | 36.09 | 30.84 | 35.09 |
|  | 36.01 | 29.32 | 36.37 |
|  | 34.56 | 30.7 |  |
|  | 40.29 | 30.82 |  |
|  |  | 30.19 |  |
|  |  | 36.35 |  |
|  |  | 34.27 |  |
|  |  | 33.16 |  |
|  |  | 34.47 |  |
|  |  | 34.54 |  |
|  |  | 32.29 |  |
|  |  | 29.27 |  |
|  |  | 31.92 |  |
|  |  | 29.95 |  |
|  |  | 32.81 |  |
|  |  | 31.23 |  |
|  |  | 32.94 |  |
|  |  | 32.08 |  |
|  |  | 33.54 |  |
|  |  | 35.24 |  |
|  |  | 31.03 |  |
|  |  | 31.72 |  |
|  |  | 35.07 |  |
|  |  | 31.93 |  |
|  |  | 30.18 |  |
|  |  | 32.62 |  |
|  |  | 30.69 |  |
|  |  | 29.99 |  |
|  |  | 31.47 |  |
|  |  | 31.91 |  |
|  |  | 32.65 |  |
|  |  | 33.75 |  |
|  |  | 29.76 |  |
|  |  | 30.9 |  |
|  |  | 39.04577 |  |
|  |  | 32.75346 |  |
|  |  | 39.41154 |  |
|  |  | 46.875 |  |
|  |  | 36.91269 |  |
|  |  | 36.93577 |  |
|  |  | 37.07923 |  |
|  |  | 39.73462 |  |
|  |  | 35.72077 |  |
|  |  | 28.065 |  |
| Mean ± SD | 33.82 ± 2.68 | 32.31 ± 2.99 | 32.33 ± 1.92 |
|  |  | p > 0.05 | p > 0.05 |

|  | **Stained area (%)** | | |
| --- | --- | --- | --- |
| **28 Days post-surgery** |  |  |  |
|  | Sal | Ang II+Veh | Ang II+Bz |
|  | 42.76423 | 70.33846 | 54.58538 |
|  | 38.87192 | 60.12077 | 39.82692 |
|  | 43.25 | 77.19884 | 42.90154 |
|  | 41.55269 | 82.27923 | 49.00385 |
|  | 74.05385 | 86.20231 | 38.62769 |
|  | 89.31346 | 77.88461 | 37.98577 |
|  | 59.64077 | 66.30846 | 41.47846 |
|  | 51.07654 | 86.90462 | 38.03962 |
|  | 56.62308 | 84.45269 | 3.908846 |
|  | 48.60154 | 88.45154 | 9.037692 |
|  | 20.14615 | 78.0127 | 18.57808 |
|  | 41.79615 | 76.90654 | 27.33154 |
|  | 7.959615 | 77.79615 | 60.78 |
|  | 7.615385 | 82.07885 | 56.495 |
|  | 6.703077 | 59.69308 | 47.60538 |
|  | 6.12 | 33.78692 | 14.33423 |
|  | 6.603077 | 54.31577 | 35.37577 |
|  | 5.878461 | 52.6 | 25.35 |
|  | 5.683846 | 65.19577 | 65.55423 |
|  | 27.27538 | 45.65423 | 26.01692 |
|  | 68.97731 | 28.56192 | 10.87462 |
|  | 69.41385 | 42.04692 | 45.86231 |
|  | 85.53423 | 59.25885 | 60.98692 |
|  | 47.45346 | 59.22923 | 36.67461 |
|  | 92.51538 | 60.63115 | 44.47346 |
|  | 19.71346 | 77.95308 | 16.01769 |
|  | 32.22577 | 73.22577 | 34.90846 |
|  | 43.52308 | 43.32808 | 28.60038 |
|  | 40.56538 | 57.26884 | 29.26462 |
|  | 44.38 | 93.47731 | 30.86077 |
|  | 33.61077 | 62.91039 | 28.79962 |
|  | 46.57384 | 78.32616 | 35.57077 |
|  | 46.69923 | 69.45039 | 40.69577 |
|  | 66.79038 | 59.68731 |  |
|  | 35.77077 | 53.95961 |  |
|  |  | 46.19846 |  |
|  |  | 15.82154 |  |
|  |  | 15.46154 |  |
|  |  | 67.23577 |  |
|  |  | 77.5127 |  |
|  |  | 67.46154 |  |
|  |  | 211.3961 |  |
|  |  | 57.42577 |  |
|  |  | 98.26654 |  |
|  |  | 59.41077 |  |
|  |  | 36.04731 |  |
|  |  | 66.73692 |  |
|  |  | 47.62077 |  |
|  |  | 64.71769 |  |
|  |  | 41.99808 |  |
|  |  | 38.96577 |  |
|  |  | 16.29923 |  |
|  |  | 42.13231 |  |
|  |  | 50.71692 |  |
|  |  | 39.40538 |  |
|  |  | 35.16462 |  |
|  |  | 51.31923 |  |
|  |  | 53.46154 |  |
|  |  | 45.44192 |  |
|  |  | 54.74269 |  |
|  |  | 67.44731 |  |
|  |  | 48.48154 |  |
|  |  | 71.07115 |  |
|  |  | 48.21346 |  |
|  |  | 76.11616 |  |
|  |  | 67.41615 |  |
|  |  | 86.96346 |  |
|  |  | 74.1823 |  |
|  |  | 78.4 |  |
|  |  | 38.08577 |  |
|  |  | 27.47923 |  |
|  |  | 35.18077 |  |
|  |  | 86.13615 |  |
|  |  | 91.19731 |  |
| Mean ± SD | 41.58 ± 24.67 | 62.47 ± 26.11 | 35.65 ± 15.31 |
|  |  | ***. p< 0.001 | ###. p< 0.001 |

| **Fig 6C** | Representative sections of ventricles stained with the ROS probe DHE. |
| --- | --- |

| **Fig 7B** | **ms** | **DO** |
| --- | --- | --- |
| **28 Days post-surgery** | \| **QTc** \|  \| \| --- \| --- \| | **TBARS** |
| **Saline** |  |  |
| 1 | 117.75 | 0.15 |
| 2 | 105 | 0.15 |
| 3 | 113.4 | 0.16 |
|  |  |  |
| **Ang II+Veh** |  |  |
| 1 | 155 | 0.36 |
| 2 | 151 | 0.21 |
| 3 | 157 | 0.26 |
| 4 | 156 | 0.26 |
| 5 | 144 | ND |
|  |  |  |
| **Ang II+Bz** |  |  |
| 1 | 121 | 0.21 |
| 2 | 122 | 0.15 |
| 3 | 127 | 0.22 |
| 4 | 132.5 | 0.35 |
|  |  |  |
| **r2** | **0.4448** | |
| **p=** | **0.025** | |

| **Fig 7A** | **ms** | **pg/mL** |
| --- | --- | --- |
| **28 Days post-surgery** | \| **QTc** \|  \| \| --- \| --- \| | **IL-6** |
| **Saline** |  |  |
| 1 | 117.75 | 0.94 |
| 2 | 105 | 0.69 |
| 3 | 113.4 | 1.18 |
|  |  |  |
| **Ang II+Veh** |  |  |
| 1 | 155 | 1.93 |
| 2 | 151 | 0.95 |
| 3 | 157 | 2.04 |
| 4 | 156 | 1.54 |
| 5 | 144 | 1.22 |
|  |  |  |
| **Ang II+Bz** |  |  |
| 1 | 121 | 0.95 |
| 2 | 122 | 1.78 |
| 3 | 127 | 0.43 |
| 4 | 132.5 | 1.42 |
|  |  |  |
| **r2** | **0.3161** | |
| **p=** | **0.071** | |

| **Fig 7C** | **ms** | **U/mg** |
| --- | --- | --- |
| **28 Days post-surgery** | \| **QTc** \|  \| \| --- \| --- \| | **SOD** |
| **Saline** |  |  |
| 1 | 117.75 | 455.23 |
| 2 | 105 | 441.84 |
| 3 | 113.4 | 602.51 |
|  |  |  |
| **Ang II+Veh** |  |  |
| 1 | 155 | 505.44 |
| 2 | 151 | 415.06 |
| 3 | 157 | 475.31 |
| 4 | 156 | 334.73 |
| 5 | 144 | 468.62 |
|  |  |  |
| **Ang II+Bz** |  |  |
| 1 | 121 | 405.02 |
| 2 | 122 | 532.22 |
| 3 | 127 | 649.37 |
| 4 | 132.5 | 492.05 |
|  |  |  |
| **r2** | **0.1168** | |
| **p=** | **0.277** | |

| **Fig 8A** | **Antioxidant/oxidant ratio** | | |
| --- | --- | --- | --- |
| **28 Days post-surgery** |  |  |  |
|  | Sal | Ang II+Veh | Ang II+Bz |
|  | 2965.7 | 1413.0 | 1966.1 |
|  | 2945.6 | 1999.3 | 3524.6 |
|  | 3673.8 | 1847.3 | 2951.7 |
|  |  | 1312.7 | 1405.9 |
|  |  | 1867.0 |  |
|  |  |  |  |
| Mean ± SD | 3195.04 ± 414.78 | 1687.87 ± 304.49 | 2462.07 ± 953.99 |
|  |  | *. p < 0.05 | p > 0.05 |

| **Fig 8B** |  |  |
| --- | --- | --- |
| **28 Days**  **post-surgery** | \| **SBP (mmHg)** \|  \| \| --- \| --- \| | **Antioxid/**  **oxid ratio** |
| **Saline** |  |  |
| 1 | 126 | 2965.67 |
| 2 | 110.75 | 2945.61 |
| 3 | 120.2 | 3673.85 |
|  |  |  |
| **Ang II+Veh** |  |  |
| 1 | 160.8 | 1413.03 |
| 2 | 156.5 | 1999.34 |
| 3 | 167.75 | 1847.31 |
| 4 | 161.5 | 1312.66 |
| 5 | 152.33 | 1867.01 |
|  |  |  |
| **Ang II+Bz** |  |  |
| 1 | 126.5 | 1966.12 |
| 2 | 126.8 | 3524.62 |
| 3 | 133.6 | 2951.69 |
| 4 | 136.75 | 1405.86 |
|  |  |  |
| **r2** | **0.5556** | |
| **p=** | **0.005** | |

| **Fig 8C** |  |  |
| --- | --- | --- |
| **28 Days**  **post-surgery** | \| **QTc (ms)** \|  \| \| --- \| --- \| | **Antioxid/**  **oxid ratio** |
| **Saline** |  |  |
| 1 | 117.75 | 2965.67 |
| 2 | 105 | 2945.61 |
| 3 | 113.4 | 3673.85 |
|  |  |  |
| **Ang II+Veh** |  |  |
| 1 | 155 | 1413.03 |
| 2 | 151 | 1999.34 |
| 3 | 157 | 1847.31 |
| 4 | 156 | 1312.66 |
| 5 | 144 | 1867.01 |
|  |  |  |
| **Ang II+Bz** |  |  |
| 1 | 121 | 1966.12 |
| 2 | 122 | 3524.62 |
| 3 | 127 | 2951.69 |
| 4 | 132.5 | 1405.86 |
|  |  |  |
| **r2** | **0.5942** | |
| **p=** | **0.003** | |

| **S2A Fig** | **Survival (%)** | |
| --- | --- | --- |
| **Days post-surgery** | **Saline** | **Ang II** |
| 0 | 100 | 100 |
| 0 | 100 | 100 |
| 7 | 100 | 100 |
| 7 | 100 | 100 |
| 14 | 100 | 100 |
| 14 | 100 | 100 |
| 21 | 100 | 100 |
| 21 | 100 | 100 |
| 28 | 100 | 100 |
| 28 | 100 | 100 |
| 58 | 100 | 100 |
| 58 | 100 | 100 |

| **S1 Fig** | Flow chart showing the experimental protocol with the number of animals used. |
| --- | --- |

| **S2B Fig** | Body weight (g) | | | | | |
| --- | --- | --- | --- | --- | --- | --- |
| **Days post-surgery** | **7** | | **28** | | **58** | |
|  | Sal | Ang II | Sal | Ang II | Sal | Ang II |
|  | 21.814 | 17.702 | 22.381 | 17.868 | 21.814 | 17.702 |
|  | 19.083 | 20.831 | 20.158 | 18.827 | 19.083 | 20.831 |
|  | 19.286 | 18.038 | 21.254 | 21.382 | 22.98 | 18.038 |
|  | 18.964 | 18.491 | 19.568 | 18.914 | 22.215 | 18.98 |
|  |  | 20.449 |  | 19.235 |  | 21.575 |
|  |  | 19.466 |  | 20.142 |  | 22.093 |
| Mean ± SD | 19.79 ± 1.36 | 19.16 ± 1.29 | 20.84 ± 1.24 | 19.39 ± 1.22 | 21.52 ± 1.70 | 19.87 ± 1.88 |
|  |  | p > 0.05 |  | p > 0.05 |  | p > 0.05 |

| **S2C Fig** | **Variation of body weight (%)** | | | | | | |
| --- | --- | --- | --- | --- | --- | --- | --- |
| **Days post-surgery** | **Saline** | | | | | | Mean ± SD |
| 0 | 0 | 0 | 0 | 0 | 0 | 0 | 0.00 ± 0.00 |
| 7 | 9.550562 | 4.945055 | -0.55249 | 3.908956 | -2.73058 | 1.857746 | 2.83 ± 4.34 |
| 14 | 5.617978 | 6.043956 | -0.55249 | 3.359173 | 0.199227 | 0.700272 | 2.56 ± 2.86 |
| 28 | 5.05618 | 12.08791 | 0.55249 | 9.956567 | 5.47287 | 9.38133 | 7.08 ± 4.19 |
|  |  |  |  |  |  |  |  |

| **S2C Fig** | **Variation of body weight (%)** | | | | | | |
| --- | --- | --- | --- | --- | --- | --- | --- |
| **Days post-surgery** | **Ang II+Veh** | | | | | | Mean ± SD |
| 0 | 0 | 0 | 0 | 0 | 0 | 0 | 0.00 ± 0.00 |
| 7 | 3.030303 | 4.6875 | -2.28571 | -3.22581 | 3.157895 | -9.97634 | -0.77 ± 5.53 |
| 14 | 4.242424 | 0 | -4.57143 | -0.53763 | 4.210526 | -7.0049 | -0.61 ± 4.56 |
| 28 | 6.666667 | 1.041667 | 2.28571 | 2.688172 | 4.210526 | -4.80383 | 2.01 ± 3.86 |
|  |  |  |  |  |  |  | **. p < 0.01 |

| **S2C Fig** | **Variation of body weight (%)** | | | | | | |
| --- | --- | --- | --- | --- | --- | --- | --- |
| **Days post-surgery** | **Ang II+Bz** | | | | | | Mean ± SD |
| 0 | 0 | 0 | 0 | 0 | 0 | 0 | 0.00 ± 0.00 |
| 7 | 0 | -2.33918 | 0 | -3.50877 | -3.01553 | 0.788288 | -1.35 ± 1.82 |
| 14 | 2.906977 | 1.754386 | 6.25 | -3.50877 | 0.164291 | -3.15315 | 0.74 ± 3.73 |
| 28 | 6.976744 | 7.017544 | 8.125 | -4.67836 | 2.28417 | -0.9009 | 3.14 ± 5.15 |
|  |  |  |  |  |  |  | **. p < 0.01 |

| **S3C Fig** | Relative heart weight  (mg/g) | |
| --- | --- | --- |
| **58 Days post-surgery** |  |  |
|  | Sal | Ang II |
|  | 4.869989 | 5.37408 |
|  | 4.276675 | 4.95944 |
|  |  | 5.88422 |
| Mean ± SD | 4.57 ± 0.42 | 5.41 ± 0.46 |
|  |  | p > 0.05 |

| **S3B Fig** | ECG evaluation | | | |
| --- | --- | --- | --- | --- |
| **58 Days post-surgery** | **Heart rate (bpm)** | | **QTc interval (ms)** | |
|  | Sal | Ang II | Sal | Ang II |
|  | 458.7 | 628.6 | 71.93 | 86.84 |
|  | 493.5 | 470.3 | 69.29 | 68.72 |
|  |  | 532.8 |  | 64.19 |
| Mean ± SD | 476.10 ± 24.615 | 543.90 ± 79.73 | 70.61 ± 1.87 | 73.25 ± 11.99 |
|  |  | p > 0.05 |  | p > 0.05 |

| **S3A Fig** | Experimental design of the blood pressure overload induced by Ang II infusion. |
| --- | --- |

| **S3D Fig** | **LVEF (%)** | |
| --- | --- | --- |
| **58 Days post-surgery** |  |  |
|  | Sal | Ang II |
|  | 56.92 | 54.61 |
|  | 53.39 | 43.68 |
|  |  | 48.63 |
| Mean ± SD | 55.16 ± 2.5 | 48.97 ± 5.47 |
|  |  | p > 0.05 |

| **S4A Fig** | Representative images of blood flow at 7-. 14-. 21- and 28-days post-surgery. |
| --- | --- |

| **S4B Fig** | Blood pressure | | | |
| --- | --- | --- | --- | --- |
| **7 Days post-surgery** | **DBP (mmHg)** | | **SBP (mmHg)** | |
|  | **Sal** | **Ang II** | **Sal** | **Ang II** |
|  | 107 | 122 | 131.5 | 160.67 |
|  | 107.34 | 150.5 | 149.333 | 145.5 |
|  | 122.34 | 150.67 | 155 | 166.67 |
|  |  | 160.34 |  | 178.67 |
|  |  | 156.67 |  | 166 |
|  |  | 156 |  | 179.5 |
|  |  | 146.5 |  | 181.5 |
|  |  | 150 |  | 168 |
|  |  | 129 |  | 170 |
|  |  | 175 |  | 178 |
|  |  | 128.5 |  | 163 |
|  |  | 159 |  | 182 |
|  |  | 147.67 |  | 163 |
| Mean ± SD | 112.22 ± 8.76 | 148.60 ± 14.64 | 145.28 ± 12.26 | 169.42 ± 10.48 |
|  |  | **. p < 0.01 |  | **. p < 0.01 |

| **S4B Fig** | **DBP (mmHg)** | | | **SBP (mmHg)** | | |
| --- | --- | --- | --- | --- | --- | --- |
| **14 Days post-surgery** |  |  |  |  |  |  |
|  | **Sal** | **Ang II+Veh** | **Ang II+Bz** | **Sal** | **Ang II+Veh** | **Ang II+Bz** |
|  | 103 | 145.5 | 127 | 114.3 | 161 | 137 |
|  | 91.5 | 132.75 | 119.5 | 118.5 | 153.5 | 153 |
|  | 98.3 | 146 | 132.5 | 114.75 | 160.5 | 161.5 |
|  |  | 136 | 144.25 |  | 146 | 140 |
|  |  | 133 |  |  | 162 |  |
|  |  | 131 |  |  | 156.5 |  |
| Mean ± SD | 97.60 ± 5.78 | 137.38 ± 6.69 | 130.81 ± 10.42 | 115.85 ± 2.31 | 156.58 ± 6.09 | 147.88 ± 11.43 |
|  |  | **. p < 0.01 | **. p < 0.01 |  | **. p < 0.01 | **. p < 0.01 |

| **S4B Fig** | **DBP (mmHg)** | | | **SBP (mmHg)** | | |
| --- | --- | --- | --- | --- | --- | --- |
| **21 Days post-surgery** |  |  |  |  |  |  |
|  | Sal | Ang II+Veh | Ang II+Bz | Sal | Ang II+Veh | Ang II+Bz |
|  | 98 | 136 | 119 | 121.75 | 148.25 | 136 |
|  | 104.5 | 133.25 | 117.2 | 125.25 | 154 | 134 |
|  | 110.33 | 137.5 | 106.5 | 123.66 | 151 | 122.75 |
|  |  | 144.66 | 108 |  | 165 | 130.6 |
|  |  | 136.66 |  |  | 161 |  |
| Mean ± SD | 104.28 ± 6.17 | 137.61 ± 4.25 | 112.68 ± 6.34 | 123.55 ± 1.75 | 155.85 ± 6.98 | 130.84 ± 5.83 |
|  |  | **. p < 0.01 | ###. p < 0.001 |  | **. p < 0.01 | ###. p < 0.001 |

| **S4B Fig** | **DBP (mmHg)** | | | **SBP (mmHg)** | | |
| --- | --- | --- | --- | --- | --- | --- |
| **28 Days post-surgery** |  |  |  |  |  |  |
|  | Sal | Ang II+Veh | Ang II+Bz | Sal | Ang II+Veh | Ang II+Bz |
|  | 113.75 | 152.2 | 118 | 126 | 160 | 126.5 |
|  | 102 | 148 | 119.6 | 110.75 | 156.5 | 126.8 |
|  | 109.8 | 151 | 123 | 120.2 | 167.75 | 133.6 |
|  |  | 153.5 | 130.25 |  | 161.5 | 136.75 |
|  |  | 140 |  |  | 152.3 |  |
| Mean ± SD | 108.52 ± 5.98 | 148.94 ± 5.40 | 122.71 ± 5.44 | 118.98 ± 7.70 | 159.61 ± 5.77 | 130.91 ± 5.09 |
|  |  | **. p < 0.01 | ###. p < 0.001 |  | **. p < 0.01 | ###. p < 0.001 |

| **S5 Fig** | **Fluorescence intensity (px)** | | |
| --- | --- | --- | --- |
| **28 Days post-surgery** |  |  |  |
|  | Sal | Ang II+Veh | Ang II+Bz |
|  | 30.44 | 32.26727 | 31.0055556 |
|  | 35.032 | 31.03455 | 32.312 |
|  | 33.83455 | 30.532 | 31.608 |
|  | 35.977 | 30.498 | 34.264 |
|  |  | 32.903 |  |
|  |  | 32.75333 |  |
|  |  | 31.392 |  |
|  |  | 37.25339 |  |
| Mean ± SD | 33.82 ± 2.42 | 32.33 ± 2.20 | 32.30 ± 1.42 |
|  |  | p > 0.05 | p > 0.05 |
|  |  |  |  |
|  |  |  |  |
| **S5 Fig** | **Stained area (%)** | | |
| **28 Days post-surgery** |  |  |  |
|  | Sal | Ang II+Veh | Ang II+Bz |
|  | 54.57481 | 78.014021 | 38.4842 |
|  | 13.57811 | 56.23965 | 28.9318 |
|  | 72.77885 | 66.560886 | 38.8455 |
|  | 40.98527 | 52.532096 | 32.5866 |
|  |  | 106.6248 |  |
|  |  | 45.026111 |  |
|  |  | 51.474884 |  |
|  |  | 66.11573 |  |
| Mean ± SD | 45.48 ± 24.94 | 65.32 ± 19.71 | 34.71 ± 4.80 |
|  |  | p > 0.05 | *. p < 0.05 |

| **S6A Fig** | Representative images and graphs from FACS analysis of CBA and concentration of the cytokines in serum studied at 28 dps.. |
| --- | --- |

**Serum cytokines**

| **Serum cytokines** | **IL-12 (pg/mL)** | | | **IL-10 (pg/mL)** | | | **TNF (pg/mL)** | | |
| --- | --- | --- | --- | --- | --- | --- | --- | --- | --- |
| **28 Days post-surgery** |  |  |  |  |  |  |  |  |  |
|  | Sal | Ang II+Veh | Ang II+Bz | Sal | Ang II+Veh | Ang II+Bz | Sal | Ang II+Veh | Ang II+Bz |
|  | 2.46 | 5.41 | 3.59 | 4.24 | 9.05 | 3.17 | 7.91 | 8.95 | 9.46 |
|  | 11.48 | 6.83 | 3.05 | 16.55 | 13.49 | 5.06 | 14.19 | 10.79 | 7.6 |
|  | 3.65 | 4.75 | ND | 5.62 | 7.63 | ND | 9.04 | 7.52 | ND |
|  | 10.4 | 8.43 | ND | 18.86 | 9.67 | ND | 18.11 | 10.33 | ND |
|  |  | 1.87 |  |  | 1.68 |  |  | 5.2 |  |
|  |  | 37.42 |  |  | 27.76 |  |  | 22.99 |  |
|  |  | 51.42 |  |  | 44.58 |  |  | 36.03 |  |
|  |  | 37.17 |  |  | 43.43 |  |  | 31.12 |  |
| Mean ± SD | 7.00 ± 4.60 | 19.16 ± 19.50 | 3.32 ± 0.38 | 11.32 ± 7.46 | 19.66 ± 16.78 | 4.12 ± 1.34 | 12.31 ± 4.73 | 16.62 ± 11.79 | 8.53 ± 1.32 |
|  |  |  | p > 0.05 | p > 0.05 | p > 0.05 | p > 0.05 | p > 0.05 | p > 0.05 | p > 0.05 |

**Heart ventricles (VD+VE)**

| **S6B Fig** | **IL-6 (pg/mL)** | | |
| --- | --- | --- | --- |
| **28 Days post-surgery** |  |  |  |
|  | Sal | Ang II+Veh | Ang II+Bz |
|  | 0.94 | 1.928214 | 0.94835 |
|  | 0.69 | 0.950606 | 1.784221 |
|  | 1.18 | 2.03701 | 0.430085 |
|  |  | 1.538582 | 1.415359 |
|  |  | 1.216492 |  |
| Mean ± SD | 0.94 ± 0.34 | 1.53 ± 0.46 | 1.14 ± 0.59 |
|  |  | p > 0.05 | p > 0.05 |

| **S6C Fig** | **SOD activity (U/mg of tissue)** | | |
| --- | --- | --- | --- |
| **28 Days post-surgery** |  |  |  |
|  | Sal | Ang II+Veh | Ang II+Bz |
|  | 455.2301 | 505.4393 | 405.0209 |
|  | 441.841 | 415.0628 | 532.2176 |
|  | 602.5105 | 475.3138 | 649.3724 |
|  |  | 334.728 | 492.0502 |
|  |  | 468.6192 |  |
| Mean ± SD | 499.86 ± 89.15 | 439.83 ± 67.18 | 519.67 ± 101.47 |
|  |  | p > 0.05 | p > 0.05 |

| **S6D Fig** | **TBARS levels (OD 532nm)** | | |
| --- | --- | --- | --- |
| **28 Days post-surgery** |  |  |  |
|  | Sal | Ang II+Veh | Ang II+Bz |
|  | 0.1535 | 0.3577 | 0.206 |
|  | 0.15 | 0.2076 | 0.151 |
|  | 0.164 | 0.2573 | 0.22 |
|  |  | 0.255 | 0.35 |
|  |  | 0.251 |  |
| Mean ± SD | 0.16 ± 0.01 | 0.27 ± 0.06 | 0.23 ± 0.08 |
|  |  | *. p < 0.05 | p > 0.05 |
